# Hippocampal output suppresses orbitofrontal cortex schema cell formation

**DOI:** 10.1038/s41593-025-01928-z

**Published:** 2025-04-14

**Authors:** Wenhui Zong, Jingfeng Zhou, Matthew P. H. Gardner, Zhewei Zhang, Kauê Machado Costa, Geoffrey Schoenbaum

**Affiliations:** 1 Intramural Research Program of the National Institute on Drug Abuse, Baltimore, MD USA; 2 State Key Laboratory of Cognitive Neuroscience and Learning, Beijing Normal University & Chinese Institute of Brain Research, Beijing, China; 3 Concordia University, Montréal, Québec Canada; 4 University of Alabama Birmingham, Birmingham, AL USA

**Keywords:** Cognitive neuroscience, Reward

## Abstract

Both the orbitofrontal cortex (OFC) and the hippocampus (HC) are implicated in the formation of cognitive maps and their generalization into schemas. However, how these areas interact in supporting this function remains unclear, with some proposals supporting a serial model in which the OFC draws on task representations created by the HC to extract key behavioral features and others suggesting a parallel model in which both regions construct representations that highlight different types of information. In the present study, we tested between these two models by asking how schema correlates in rat OFC would be affected by inactivating the output of the HC, after learning and during transfer across problems. We found that the prevalence and content of schema correlates were unaffected by inactivating one major HC output area, the ventral subiculum, after learning, whereas inactivation during transfer accelerated their formation. These results favor the proposal that the OFC and HC operate in parallel to extract different features defining cognitive maps and schemas.

## Abstract (web summary)

Inactivation of the ventral subiculum does not change existing schema cells in the orbitofrontal cortex (OFC) but aids their formation during new learning. This challenges the idea that the OFC relies on the hippocampus and suggests that these regions work in parallel to process different aspects of cognitive maps.

## Main

The orbitofrontal cortex (OFC) and hippocampus (HC) are both associated with the process of forming mental constructs—cognitive maps^1,2^—that permit adaptive behavior in situations where novelty or incomplete information prevents reliance on past experience^3–5^. Although first applied to explain the role of the HC in mapping space and other informational dimensions in relational memory, the same term accurately describes the involvement of the OFC in sussing out the components and relationships that define the world around us, particularly as relevant to our behavioral goals or purpose in a particular setting. Accurate knowledge of such task spaces—composed of the internally specified states and state transitions that comprise the task at hand^6,7^—can be enormously useful, whether navigating a maze to obtain pellets, a metro system to reach the airport or social structures to get ahead. Having an accurate task map allows us to rapidly recognize new or incomplete information and then respond in a manner consistent with our needs and desires. This principle extends to the formation of schemas, which we would define as generalized cognitive maps, and facilitates the transfer of knowledge to new problems of a similar type, as when knowledge of one metro system makes it easier to learn to use another. Although schemas can cause difficulties when applied inappropriately, they normally facilitate ongoing behavior, because they provide shortcuts for responding in new situations. Notably, neural activity reflecting schema formation has been demonstrated in both the OFC and the HC^8–11^.

This convergence in function puts renewed emphasis on understanding how the two areas interact. Historically, addressing this has been hampered by the very different tasks used to study the HC, which typically focus on spatial information and navigation, versus those applied to the OFC, which normally use nonspatial sensory modalities, especially chemosensory, in simpler Pavlovian or instrumental tasks. Notable exceptions to this dichotomy have shown that the OFC maps spatial relationships in settings normally used to assess HC function^12–15^ and that the HC reflects information and contributes to adaptive behaviors more normally associated with the OFC^16–22^. When neural activity in the two areas is directly compared in the same task, similarities and differences are evident^10,11,23–28^. Both areas construct a model of the task space, but the OFC appears to give precedence to biologically significant information, whereas the HC represents externally defined states with greater fidelity, even when incidental to task performance^10,27,28^.

Yet, although such comparative studies provide glimpses into how the two areas may interact in the formation of cognitive maps and schemas, they are usually conducted at steady state rather than during learning, rarely address transfer to new problems and generally do not test for effects of manipulations of one area on correlates in the other. As a result, current evidence can be used to support either serial or parallel processing models^10,27–33^. For instance, the HC may build a task map or schema based on external stimuli, which is then accessed by areas such as the OFC for extraction of behaviorally relevant features, both within and across problems. In this scenario, task representations in the OFC would be heavily dependent on HC processing. Alternatively, the OFC and HC may function in parallel, extracting different information relevant to task mapping and schema formation according to each area’s unique functions. Under this arrangement, many features of the representations in the OFC would be independent of the HC.

In the present study, we tested between the predictions of these two models, asking specifically how generalized representations—schemas—encoded in single-unit activity in the OFC, are affected by inactivation of the ventral subiculum, a major outflow pathway of the HC that projects strongly to the OFC, both during performance on well-learned problems and during transfer to new problems. Our results distinguish between the two alternative models, strongly favoring the proposal that the OFC and HC operate in parallel to extract different features defining cognitive maps and schemas during the integration of new information.

## Results

**Fig. 1:**
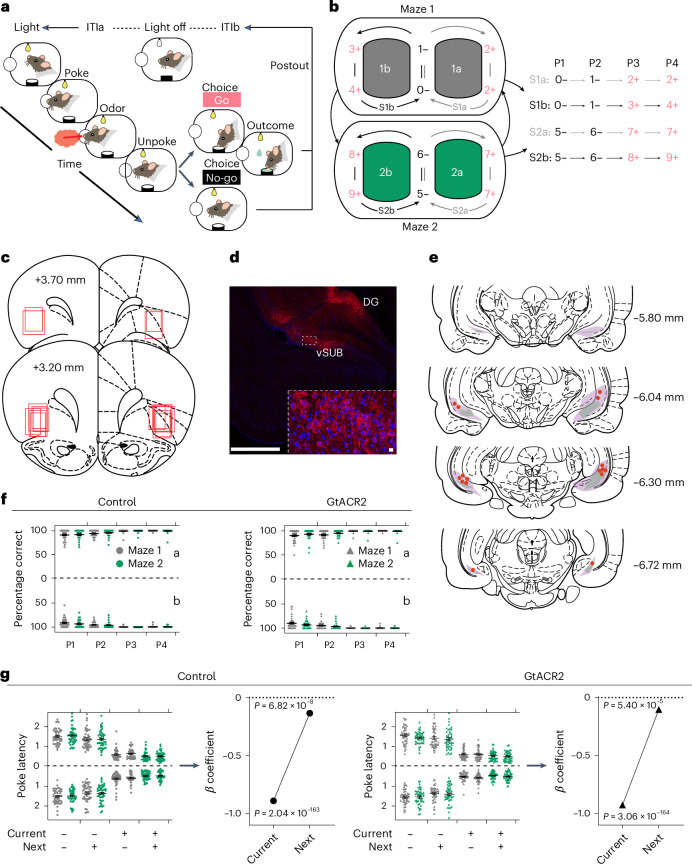
Task design, histology and behavior. **a**, Schematic illustrating the events of a trial in the odor-sequence task. The illumination of two overhead house lights indicated the start of each trial. After poking into the central odor port and sampling the presented odor, rats could respond with a ‘go’ to obtain a sucrose reward or a ‘no-go’ to avoid a prolonged intertrial interval. **b**, Odor-sequence task illustrated as two virtual figure-of-eight mazes. Ten odors were organized into two sequence pairs (S1 and S2), each comprising two subsequences (**a** and **b**). Each subsequence consists of four trials or positions (P1–P4) indicated by odor numbers. Red +, rewarded; black −, nonrewarded; 0–9, odor identities; arrows indicate sequence transitions. **c**, Reconstruction of recording locations in the lateral OFC. The approximate extent of recording locations in each rat is represented by red squares. **d**, Virus expression. An adeno-associated virus (AAV) carrying the soma-targeted GtACR2-FusionRed construct under the CaMKIIa promoter was injected into the ventral subiculum (vSub) bilaterally. GtACR2-expressing neurons were identified using immunohistochemistry (red, GtACR2; blue, DAPI). GtACR2-expressing neurons were found in the vSUB and dentate gyrus (DG). This experiment was independently repeated across all eight animals, yielding consistent results. Individual neurons expressing GtACR2 are magnified in the dashed white box. Scale bars, 1 mm (left) and 10 µm (right). **e**, Reconstruction of GtACR2 expression and optical fiber placements in the vSUB. The maximal and minimal extents of GtACR2 expression are indicated by purple and green colors, respectively, and red dots indicate optical fiber placement. **f**,**g**, Percentage correct (**f**) and latency to poke into the odor port to initiate a trial after light onset (**g**) on each trial type in S1a, S2a (above *y* axis), S1b, S2b (below *y* axis) for control (left) and GtACR2 (right) sessions (gray, maze 1; green, maze 2). The error bars represent the s.e.m. Four-way ANOVAs confirmed the significant main effects of position on both measures (percentage correct: *F*_(3,1405)_ = 145.5, *P* = 4.2 × 10^−82^, ${{{\eta }}}_{{\rm{p}}}^{2}$ = 0.24; poke latency: *F*_(3,1405)_ = 889.1, *P* = 1.0 × 10^−^^323^, ${{{\eta }}}_{{\rm{p}}}^{2}$ = 0.66; *n* = 45 sessions for control; *n* = 44 sessions for GtACR2), with reward driving more accurate and faster performance. Further regression analyses on the latency to initiate trials showed that this measure was affected by whether the reward was to be delivered on both the current and the next trials (**g**, right for control and GtACR2). Notably, in these analyses, there were no effects of inactivation (*F* < 0.82; *P* > 0.36; ${{{\eta }}}_{{\rm{p}}}^{2}$ < 0.0006; *n* = 45 sessions for control; *n* = 44 sessions for GtACR2). The error bars are the s.e.m. (see Extended Data Fig. 1). Source data

Single-unit activity was recorded in the OFC in rats (*n* = 4 females, 4 males) performing an odor-sequence task built on top of a standard go or no-go odor discrimination (Fig. 1a). In each trial, the rats sampled an odor presented at a centrally located port and then had to decide whether to respond at a nearby fluid well for a sucrose reward. Rather than being randomized, however, the odor cues presented on successive trials were arranged in a predictable, fixed sequence to define trajectories through a virtual ‘figure-of-eight’ maze. In initial training, 10 different odor cues were arranged to form two unique figure-of-eight mazes, with similar reward structures (Fig. 1b) and rats performed two alternating 80-trial blocks of each maze in each session. Critically, the rats could use the odor cues on each trial to correctly respond for reward, but they could also use the sequence to anticipate reward many trials into the future, like a rat traveling through a sequence of positions on an actual figure-of-eight maze.

**Extended Data Fig. 1:**
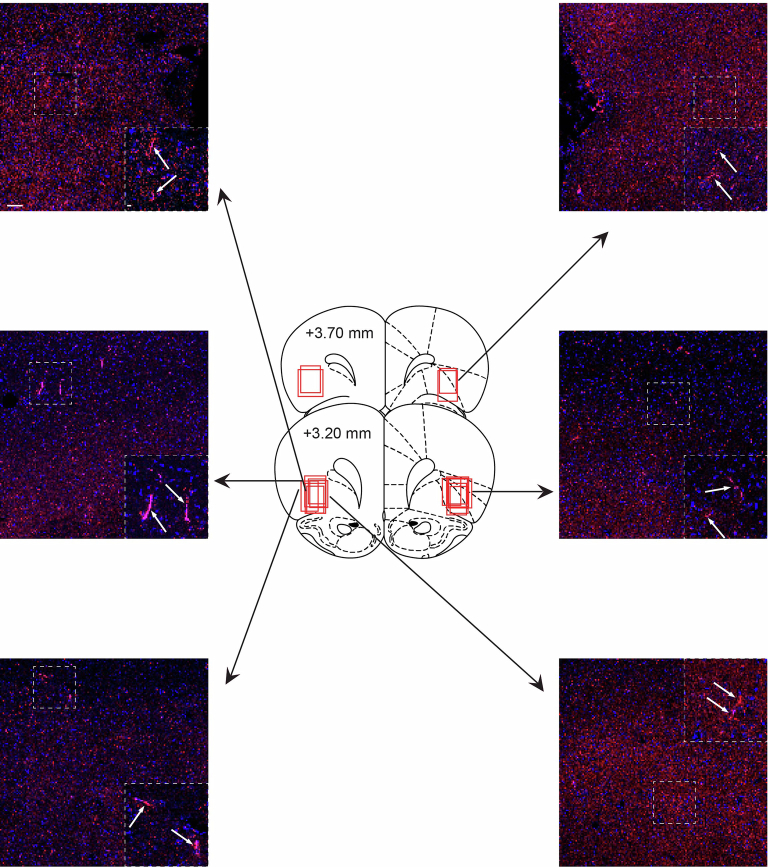
Histology. Reconstruction illustrating the recording sites within the lateral orbitofrontal cortex (OFC) containing axons from transfected VSub neurons, expressing GtACR2. The approximate extent of recording sites in each rat is depicted by red squares. This experiment was independently repeated with all six animals, producing consistent results. OFC axons from VSub neurons expressing GtACR2 are showing a higher magnification within the dashed white box and indicated by white arrows. Scale bars are provided at 100 µm and 10 µm.

Once rats were trained to perform the task, electrodes were implanted in the OFC to allow single-unit recording and fibers were implanted over the ventral subiculum after infusion of pAAV-CKIIa-stGtACR2-FusionRed^34^ (Addgene viral prep, cat. no. 105669-AAV1) to allow inactivation of hippocampal outflow (Fig. 1c–e). Axons originating from ventral subiculum neurons expressing GtACR2 were consistently observed within the lateral OFC across all the rats in our study (Extended Data Fig. 1). Recording began 5–6 weeks after recovery from surgery and retraining on the task while tethered. During recording, each rat completed sessions in which 465-nm light was delivered to activate GtACR2, thereby inactivating the ventral subiculum, during each trial. Each inactivation session was accompanied by a reminder session and a second recording session at the same location, during which light of an ineffective wavelength (630 nm) was delivered to serve as a control^35^. The order of these three sessions was counterbalanced such that control and inactivation sessions at each location were equally likely to be preceded by either a control or a retraining session, avoiding a situation in which control sessions directly followed inactivation (see Supplementary Fig. 1 for illustration of design). In both inactivation and control sessions, rats maintained highly accurate discrimination performance at all positions in both mazes (Fig. 1f) and showed differences in their latencies to initiate trials, indicating the use of the sequences to predict, at the start of each trial, whether reward would be delivered on that trial and the next trial (Fig. 1g). There were no effects of maze or inactivation (see figure captions for statistics).

**Fig. 2:**
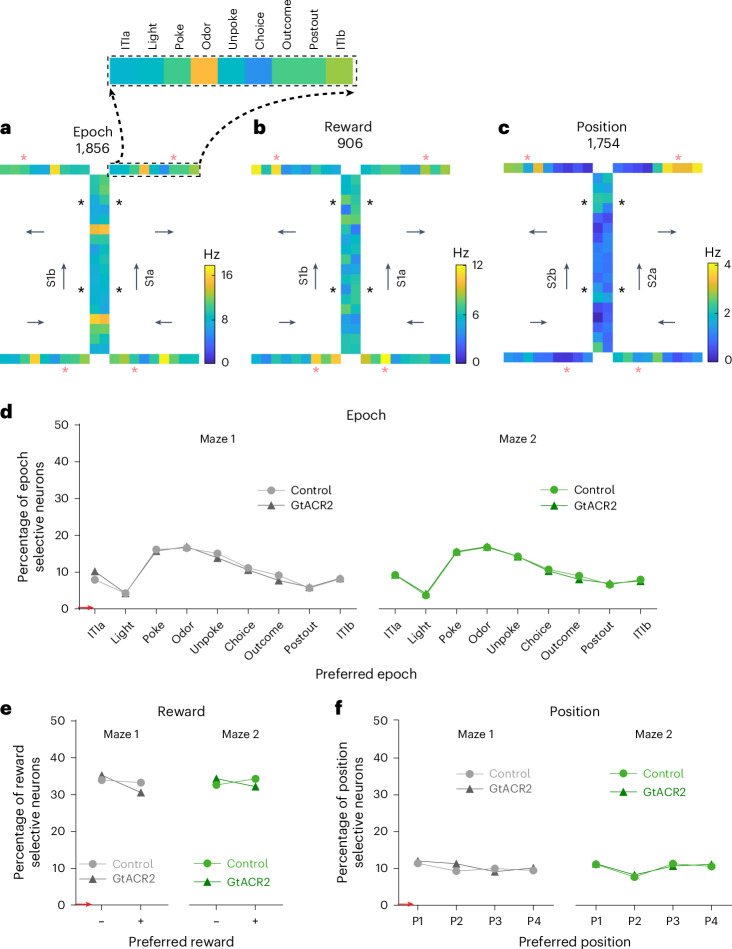
Exemplar units illustrating the influence of epoch, reward, position and quantification across the population. **a**–**c**, Heatmaps of the OFC neurons showing epoch-specific (**a**), reward-specific (**b**) and position-specific (**c**) firing in the figure-of-eight task. In each panel, the heatmap shows average activity in each epoch at each position in one maze. Individual squares corresponding to each epoch are magnified in the black dashed box at the top. Arrows represent sequence directions. A red asterisk marks the reward epoch on rewarded trial types (P3 and P4), whereas a black asterisk marks the reward epoch for nonrewarded trial types (P1 and P2). **d**–**f**, Plots show the percentage of the OFC neurons with firing that was significantly modulated by epoch (**d**), reward (**e**) and position (**f**) (ANOVA, *P* < 0.01), with each neuron assigned to the condition of maximal firing. There were no effects of inactivation (*χ*^2^ < 1.42; *P* > 0.23; degree of freedom (d.f.) = 1; *χ*^2^ test). Red denotes the chance level.

We recorded a total of 1,856 units in the OFC during the control sessions and 1,834 units during the inactivation sessions. To visualize the patterns of firing during task performance, we calculated the activity of each single unit during each of nine epochs tied to the specific events spanning each trial (intertrial interval a (ITIa), light, poke, odor, unpoke, choice, outcome, postoutcome, intertrial interval b (ITIb)) for each of the eight positions in each maze. This analysis revealed a great variety of patterns; however, the activity of individual units was generally influenced by some combination of trial epoch, reward and maze position (Fig. 2a–c). Overall, single-unit activity in the OFC was significantly influenced by each of these variables, with no apparent effect of inactivation (Fig. 2d–f).

**Fig. 3:**
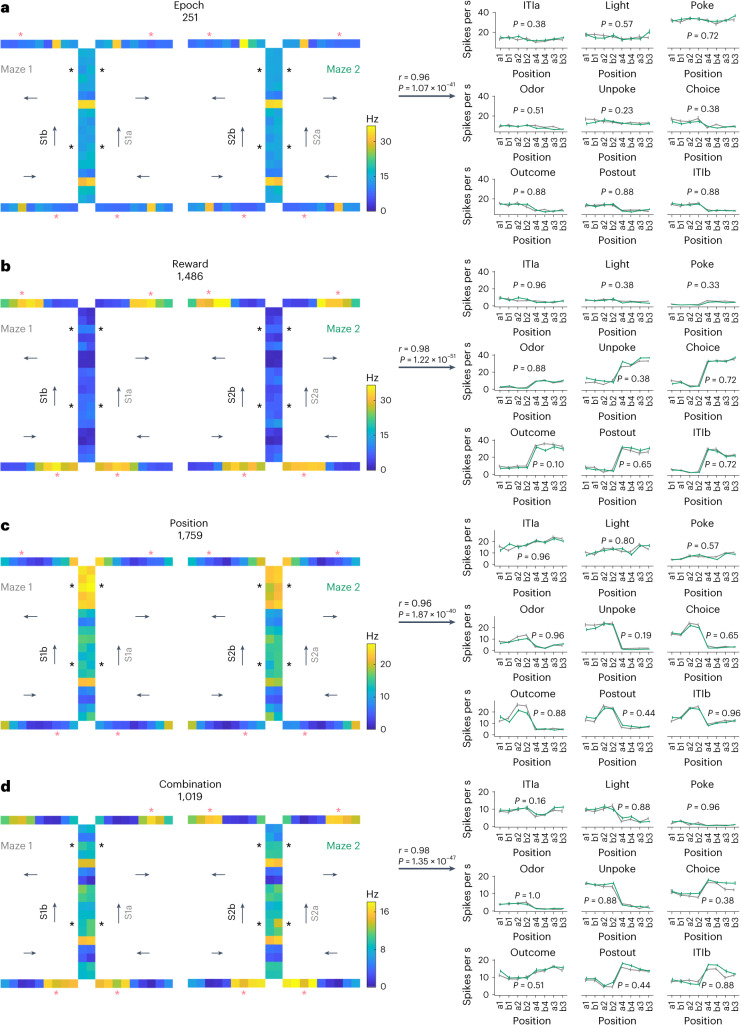
Exemplar units illustrating the generalization of epoch, reward and positional information across mazes. **a**–**d**, Heatmaps (left) and mean firing rate at each position and epoch (right) for OFC neurons showing generalization of activity related to epoch (**a**), reward (**b**) and position (**c**) or a combination of factors (**d**). Heatmaps plot activity as described in Fig. 2. Line plots show the average firing rate in each epoch at each position in each maze, ordered according to the reward on the current and next trials. The gray line represents maze 1 and the green line maze 2. The firing rates were not significantly different between maze 1 and maze 2 at all epochs in each example (*P* > 0.10; two-sided Wilcoxon’s rank-sum test; *n* = 8 positions for each maze of each neuron). The error bars are the s.e.m. (Extended Data Figs. 2–4).

**Extended Data Fig. 2:**
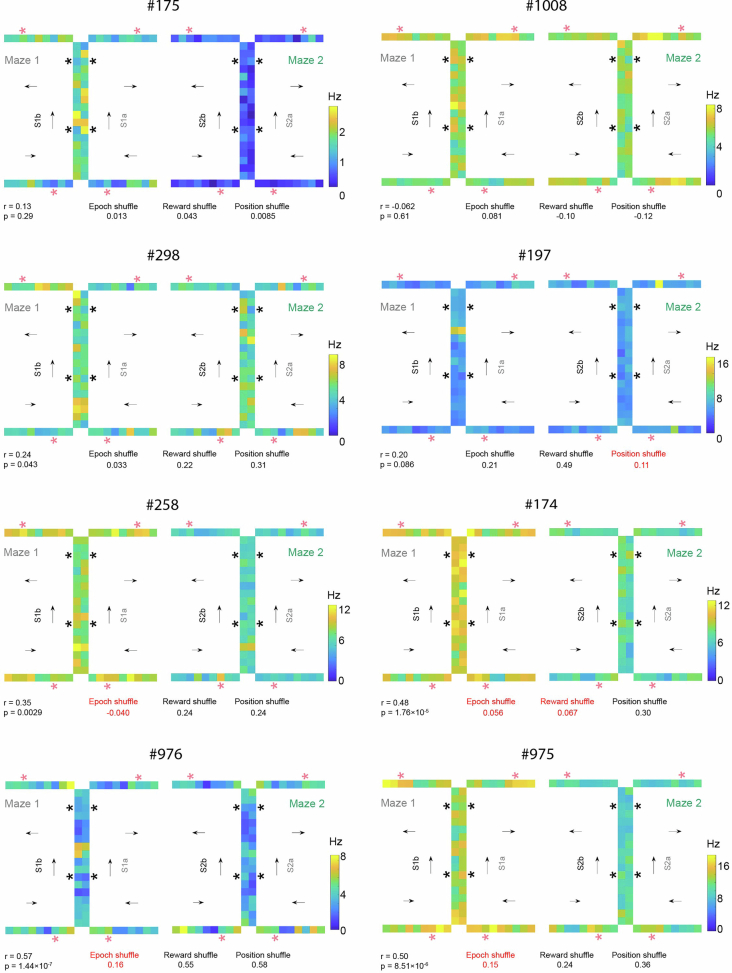
Exemplar units illustrating maze-unique (that is non-generalized) firing patterns in OFC. A pair of heatmaps are shown for each neuron, plotting average activity in each epoch at each position in each maze. Arrows represent sequence directions. Red * marks the reward epoch on rewarded trial types (P3 and P4), while black * marks the reward epoch for non-rewarded trial types (P1 and P2). The correlation coefficients before and after shuffling of epoch, reward, and position are presented in the lower panel of each figure; numbers shown in red were significantly affected by shuffling.

**Extended Data Fig. 3:**
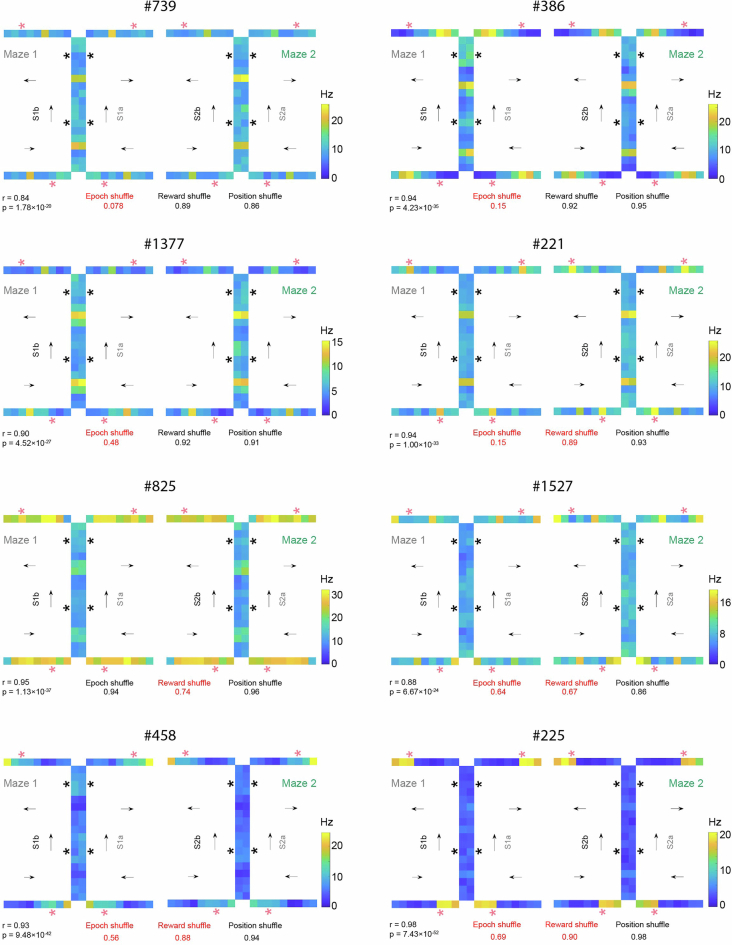
Exemplar units illustrating the generalization of epoch and reward information across mazes in OFC. A pair of heatmaps are shown for each neuron, plotting average activity in each epoch at each position in each maze. Arrows represent sequence directions. Red * marks the reward epoch on rewarded trial types (P3 and P4), while black * marks the reward epoch for non-rewarded trial types (P1 and P2). The correlation coefficients before and after shuffling of epoch, reward, and position are presented in the lower panel of each figure; numbers shown in red were significantly affected by shuffling.

**Extended Data Fig. 4:**
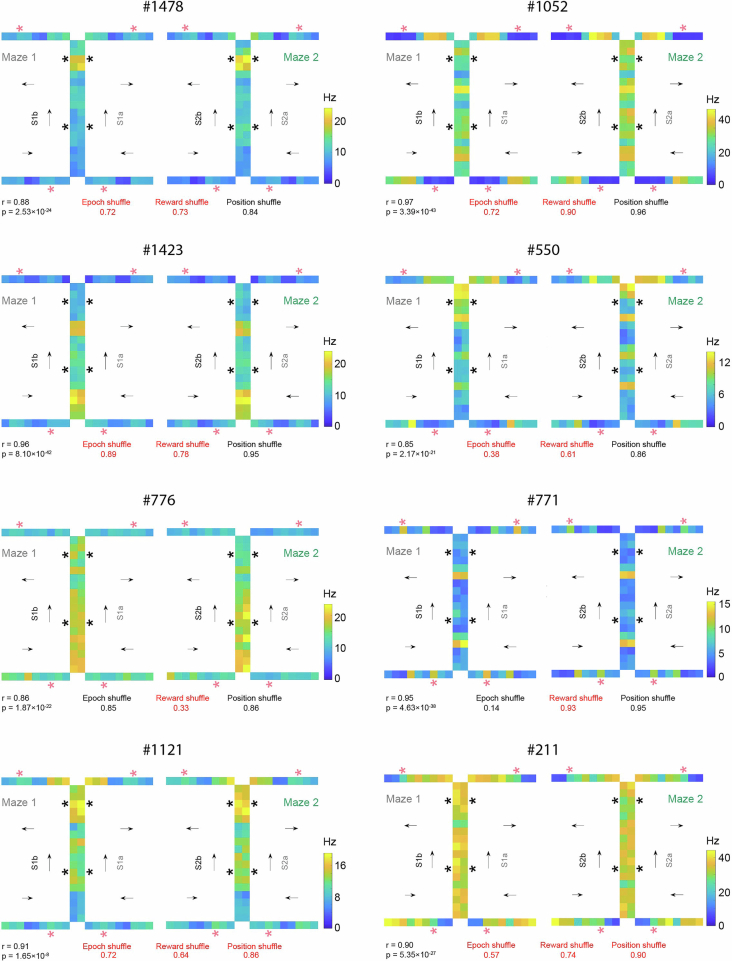
Exemplar units illustrating the generalization of positional information across mazes in OFC. A pair of heatmaps are shown for each neuron, plotting average activity in each epoch at each position in each maze. Arrows represent sequence directions. Red * marks the reward epoch on rewarded trial types (P3 and P4), while black * marks the reward epoch for non-rewarded trial types (P1 and P2). The correlation coefficients before and after shuffling of epoch, reward, and position are presented in the lower panel of each figure; the numbers shown in red were significantly affected by shuffling.

Importantly, although the activity of some units differed between the two mazes (Extended Data Fig. 2), many showed very similar discrete firing patterns across them, consistent with representation of a generalized cognitive map or schema of the virtual figure-of-eight task. The generalization of the representations across the two mazes typically reflected the influence of the same variables noted above to impact unit firing, specifically trial epoch (Fig. 3a), reward (Fig. 3b), position (Fig. 3c) or some combination of these factors (Fig. 3d and Extended Data Figs. 3 and 4). Although the generalization of variables such as epoch or reward would not necessarily require recognition of the common structure between the two mazes (for example, see examples in Fig. 3a,b), in other cases generalization required recognition of this arbitrary structure. For example, in some units, activity was driven by the meaning of specific positions within the sequence (for example, the cell in Fig. 3c, which fired most at P2 during choice → ITIb epochs) and, in others, it appeared to reflect still more idiosyncratic information characterizing particular epochs and positions (for example, the cell in Fig. 3d, which fired at rewarded positions and unpoke at P1 and P2).

### Ventral subiculum inactivation does not affect prevalence or content of schema cells in the OFC on an established problem

To quantify the various patterns observed in the single-unit correlates, we designed an algorithmic set of correlational analyses to categorize each unit as representing trial structure, reward or position and to assess the generalization of that information across mazes. For each unit, we calculated the actual mean firing across all trials in each of the nine epochs at each of the eight positions in the two mazes, yielding a pair of 72 × 1 data arrays (that is, nine epochs at each of eight positions). We then used correlation coefficients on the resultant pair of 72 × 1 data arrays to determine the generalizability of activity across the two mazes, defining as a schema cell any unit that exhibited a very strong significant correlation (*r* > 0.8 and *P* < 0.01)^36^. This approach was modeled on recent work by Baraduc et al.^9^ to identify schema cells in the HC of monkeys performing a similar virtual maze task. We also defined each unit as influenced by epoch, reward or position if the correlation across mazes was significant and survived shuffling of information in the other two dimensions (see Methods for description). This approach allowed us to classify the activity of each single unit as influenced by each type of information independently, so that we could assess the interrelationship of the units, their generalization and any effects of time and inactivation.

**Fig. 4:**
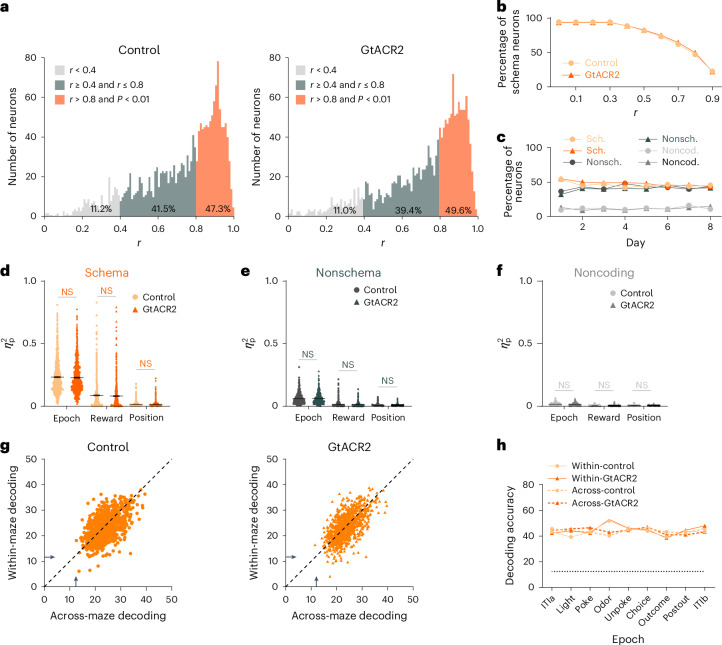
Ventral subiculum inactivation does not affect the prevalence or positional decoding of schema cells in the OFC during performance on an established problem. **a**, Correlation in firing across mazes for all OFC neurons recorded in control and GtACR2 sessions. The plots show the distribution of *r* scores with the neurons that met the arbitrary cutoff for classification as schema cells (*r* > 0.8, *P* < 0.01, correlation coefficients) shown in orange, nonschema cells (*r* ≥ 0.4 and *r* ≤ 0.8, correlation coefficients) in dark gray and noncoding cells (*r* < 0.4, correlation coefficients) in light gray. **b**, Percentage of schema neurons at different thresholds for categorization. There was no difference between the two groups in the proportion of neurons at any threshold value (*χ*^2^ = 0.67; *P* = 0.41; d.f. = 1; *χ*^2^ test). **c**, Percentage of schema (Sch.), nonschema (Nonsch.) and noncoding (Noncod.) neurons from control and GtACR2 sessions on each day of training (using thresholds in **a**). There was no difference between the two groups in the proportion of neurons at any day (*χ*^2^ < 2.1; *P* > 0.15; d.f. = 1; *χ*^2^ test). **d**–**f**, Explained variance, averaged across neurons, for each factor (epoch, reward, position) within maze in the schema (**d**; *n* = 877 units for control; *n* = 910 units for GtACR2), nonschema (**e**; *n* = 771 units for control; *n* = 722 units for GtACR2) and noncoding (**f**; *n* = 208 units for control; *n* = 202 units for GtACR2) populations. There were no effects of inactivation (*P* > 0.09; two-tailed Student’s *t*-test). **g**, Accuracy of decoding position across all epochs by individual schema cells, where → denotes chance decoding of 12.5%. One-way ANOVA showed that accuracy was similar for decoding within and across mazes for neurons in control (*F*_(1,3710)_ = 0.068; *P* = 0.79; ${{{\eta }}}_{{\rm{p}}}^{2}$ = 1.8 × 10^−5^) and GtACR2 sessions (*F*_(1,366)_ = 0.016; *P* = 0.90; ${{{\eta }}}_{{\rm{p}}}^{2}$ = 4.4 × 10^−6^) and there was no significant effect of inactivation (within: *F*_(1,368)_ = 0.45; *P* = 0.50; ${{{\eta }}}_{{\rm{p}}}^{2}$ = 1.2 × 10^−4^; across: *F*_(1,3688)_ = 0.35; *P* = 0_._55; ${{{\eta }}}_{{\rm{p}}}^{2}$ = 9.5 × 10^−5^). **h**, Accuracy of decoding position within each epoch by ensembles of schema cells, where the dotted line denotes chance decoding of 12.5%. A one-way ANOVA showed that accuracy was similar for decoding within and across mazes for neurons in control (*F*_(1,16)_ = 0.02; *P* = 0.88; ${{{\eta }}}_{{\rm{p}}}^{2}$ = 1.5 × 10^−3^) and GtACR2 sessions (*F*_(1, 16)_ = 0.31; *P* = 0.58; ${{{\eta }}}_{{\rm{p}}}^{2}$ = 0.02) and there was no significant effect of inactivation (within: *F*_(1,16)_ = 0.37; *P* = 0.55; ${{{\eta }}}_{{\rm{p}}}^{2}$ = 0.023; across: *F*_(1, 16)_ = 0; *P* = 0.96; ${{{\eta }}}_{{\rm{p}}}^{2}$ = 1.6 × 10^−4^; Supplementary Figs. 2–5 and 10).

The analysis identified generalized representations of the two mazes in nearly half of the units recorded in control sessions (Fig. 4a left, orange shading). Inspection of heat plots showing the activity of each unit confirmed that these neurons generally had very similar firing patterns in each maze (similar to examples in Fig. 3 and Extended Data Figs. 3 and 4). Units that failed to meet these stringent criteria and had correlations <0.4 tended to have little phasic firing in the task (Fig. 4a, left, light-gray shading), so they were categorized as noncoding, whereas units with correlations between 0.4 and 0.8 were categorized as nonschema cells (Fig. 4a, left, dark-gray shading, and Extended Data Fig. 2). Notably, the proportions in each category were nearly identical to the proportions recorded at the same locations in these rats when the ventral subiculum was inactivated in other sessions (Fig. 4a, right). Importantly, although the correlation used to categorize a unit as a schema cell was based on a somewhat arbitrary criterion, the lack of any effect of inactivation was true regardless of the precise threshold (Fig. 4b); similarly, nonschema and noncoding neurons exhibited comparable results (Supplementary Fig. 2) and the proportion of cells that fell in each category was remarkably stable across sessions and repeated inactivation (Fig. 4c). An analysis to determine the average explained variance related to epoch, reward and position indicated that this information was more prevalent in schema than in nonschema cells, and there was no impact of inactivation on any category (Fig. 4d–f and Supplementary Fig. 3).

Schema cells identified in this manner exhibited relatively high and similar classification performance within versus across the two mazes. To show this for the individual cells, we used activity across epochs at each position in one maze as the training set for classification of trials drawn at random from either that maze or the other maze on which that neuron was characterized in a particular session. Using this approach, activity from individual OFC neurons correctly classified the position of test trials ~12.5% of the time, on average. This performance did not depend on whether the test trial came from the same or the opposite maze as the training set data and again there was no effect of inactivation (Fig. 4g). Significant distinctions in single-cell decoding were observed among schema, nonschema and noncoding neurons (Supplementary Fig. 4). We also repeated this analysis using activity from ensembles of schema cells within each epoch; ensemble activity correctly classified the position of test trials ~45% of the time. Again, this performance was similar within and across mazes and was not affected by inactivation (Fig. 4h). Moreover, significant differences were observed in cell ensembles across schema, nonschema and noncoding categories as well (Supplementary Fig. 5).

**Fig. 5:**
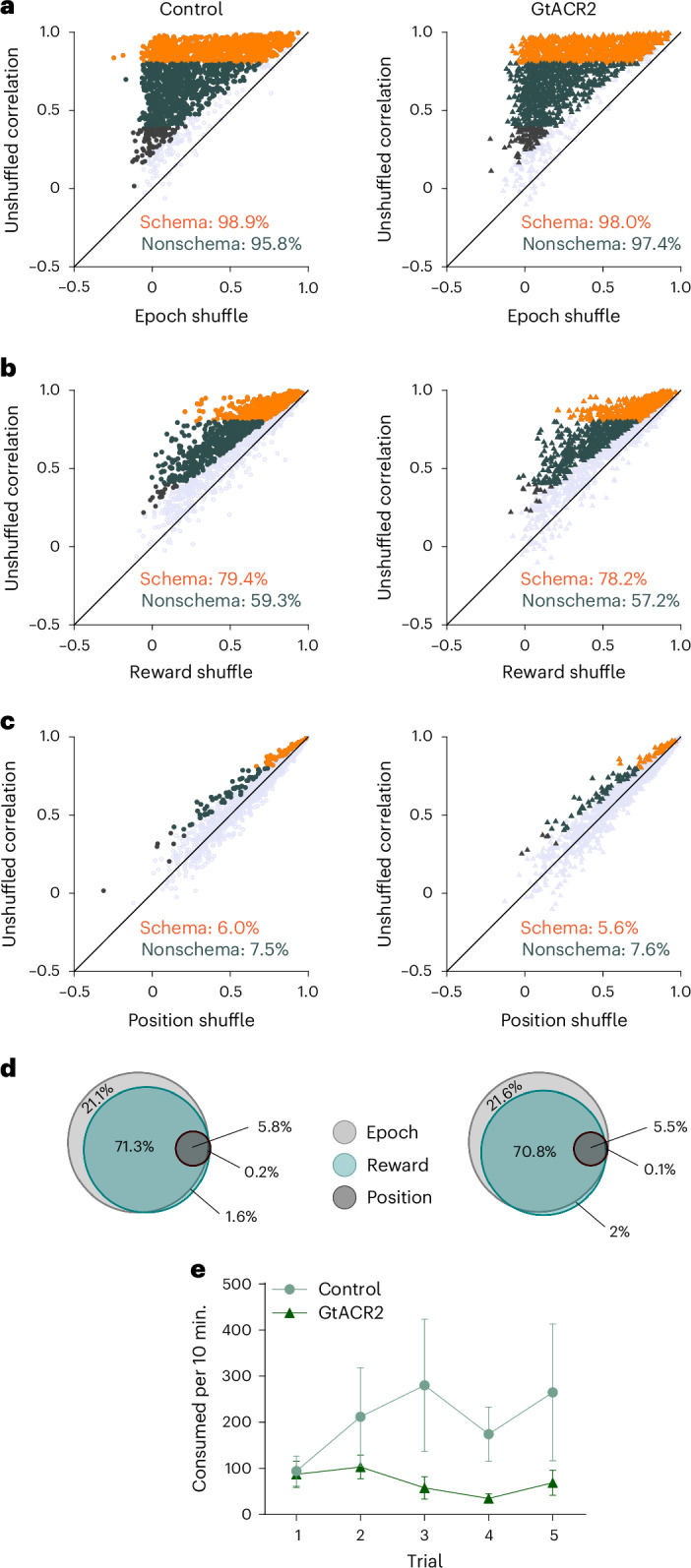
Ventral subiculum inactivation does not affect the content of schema cells in the OFC during performance on an established problem. **a**–**c**, Scatter plots showing the correlation coefficients of each neuron from the control (left) and GtACR2 (right) sessions. The *y* axes plot the correlation coefficients from unshuffled data and the *x* axes the mean correlation coefficients obtained after shuffling data (1,000×) to disrupt contributions of information related to the epoch (**a**), reward (**b**) or position (**c**). Orange, gray or black cells had actual correlation coefficients >99% of the shuffled results, indicating a significant contribution of the shuffled type of information to the correlated firing patterns. These populations, the percentage of the total of that category noted on the panels, were not affected by inactivation (*χ*^2^ < 3.4; *P* > 0.066; d.f. = 1; *χ*^2^ test). Orange denotes schema cells and gray nonschema cells. **d**, Venn diagrams summarizing data from **a** to **c**, showing the fraction of schema neurons recorded in control and GtACR2 sessions that were affected by the shuffling of information related to epoch (light gray), reward (light green) and position (dark gray). The sizes of circles are normalized to the total number of neurons recorded in each group and proportions in each category that overlap between categories were not affected by inactivation (*χ*^2^ < 0.40; *P* > 0.54; d.f. = 1; *χ*^2^ test). **e**, Food consumption across trials in the neophobia task. Lines show new food consumed per trial as a percentage of familiar food. Light green, control and deep green, GtACR2. A three-way ANOVA revealed a significant main effect of novelty (*F*_(1,104)_ = 9.11; *P* = 0.0032; ${{{\eta }}}_{{\rm{p}}}^{2}$ = 0.081; *n* = 5 trials for both groups) and a significant interaction between the novelty and group (*F*_(1,104)_ = 4.05; *P* = 0.047; ${{{\eta }}}_{{\rm{p}}}^{2}$ = 0.038; *n* = 5 trials for both groups). Further testing showed a significant difference between groups on the last three (*F*_(1,34)_ = 7.21; *P* = 0.01; ${{{\eta }}}_{{\rm{p}}}^{2}$ = 5.6 × 10^−3^; *n* = 3 trials for both groups) but not the initial two trials (*F*_(1,22)_ = 0.95; *P* = 0.34; ${{{\eta }}}_{{\rm{p}}}^{2}$ = 0.042; *n* = 3 trials for both groups). The error bars are the s.e.m.

Inactivation of the ventral subiculum also had very little effect on the content of the generalized representations in the OFC. The fractions of units with correlated activity that reflected trial epoch (Fig. 5a), reward (Fig. 5b) or position (Fig. 5c) were entirely unaffected by inactivation, as were the proportion of neurons in each of these categories that met criteria for being schema cells (orange fraction in Fig. 4a and Fig. 5d). This lack of effect was also evident when we restricted this analysis to the early periods of the trial (ITIa → odor), which were uncontaminated by external stimuli and actions related to the go or no-go responses or the presence of reward (Supplementary Fig. 6). Thus, inactivation of hippocampal outflow did not dramatically impact established correlates, generalized or not, in the OFC.

Importantly, these negative effects were obtained despite good viral expression and fiber placement (Fig. 1d,e). In addition, we paused the experiment at this point to behaviorally validate the efficacy of inactivation. Rats were trained in a food neophobia task known to depend on hippocampal memory systems^37–40^. Each day they were given a choice between a new food pellet and pellets made of their normal chow, and consumption was measured over a 10-min period, during which the rats received either light effective in inactivating the ventral subiculum (*n* = 3) or ineffective light serving as a control (*n* = 3). We reasoned that, if the appropriate wavelength light was disrupting hippocampal outflow, then the rats receiving it would have difficulty remembering prior exposures to the new pellet and would show prolonged neophobia relative to their counterparts. Consistent with this prediction, we found that controls increased their consumption of the new food relative to the familiar one, whereas the inactivated group did not (Fig. 5e). These results provide independent confirmation that light delivery in these rats was acting as expected to disrupt hippocampal output.

### Ventral subiculum inactivation facilitates formation of schema cells in the OFC during learning of new problems

**Extended Data Fig. 5:**
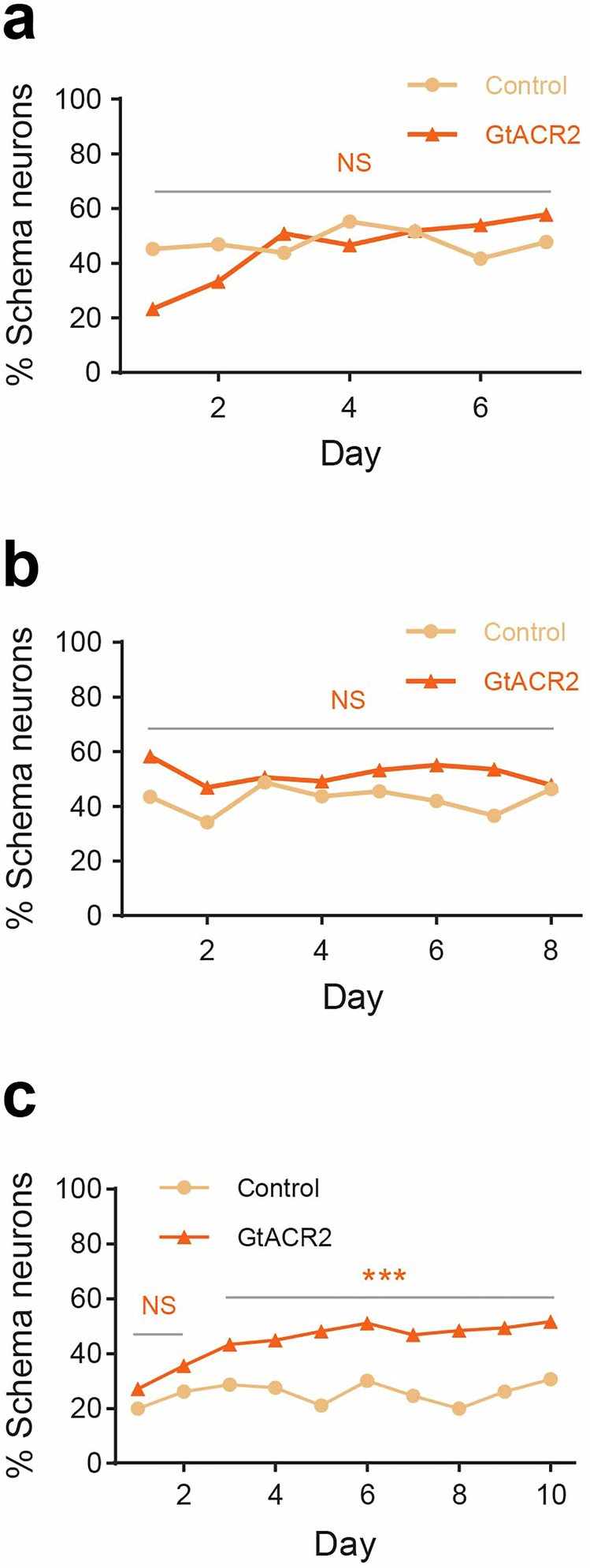
Percentage of schema neurons during prior training from the rats comprising the control and GtACR2 groups in the learning experiment (Figs. 6–8) and the impact of removal of 2 replacement rats on effect of inactivation during learning. **a-b**, Percentage of schema neurons on each day of retraining after surgery from the rats comprising the control and GtACR2 groups in the learning experiment (**a**) and from control sessions on the well-learned task (**b**). There were no differences between the rats in the two groups on any prior day of training (retraining: χ^2^ < 6.0; P > 0.10; d.f. = 1; χ^2^ test; well-learned problem: χ^2^ < 3.94; P > 0.38; d.f. = 1; χ^2^ test). False discovery rate (FDR) and Benjamini-Hochberg (BH) corrections were applied to correct for multiple comparisons. **c**, Effect of inactivation of ventral subiculum on prevalence of schema cells during learning, excluding data from the 2 replacement rats added during this phase; without data from these rats, the prevalence of schema cells in the two groups was similar initially and then diverged thereafter (Overall: χ^2^ = 84.4, P = 4.0 ×10^−20^; days 1-2: χ^2^ = 4.0; P = 0.05; d.f. = 1; χ^2^ test; days 3-10: χ^2^ = 83.5, P = 6.4 ×10^−20^; d.f. = 1; χ^2^ test). (***P < 0.0001; NS, not significant).

Next, we recorded from the OFC during learning of two new problems. The new problems were identical in structure to the first problem (Fig. 1b), except that ten new odors were used for each. Single units were recorded for 10–12 d of training on each problem (9 d of acquisition and then a final day). For this phase, the rats that completed prior training and remained healthy (*n* = 4) were divided equally into two groups, along with two additional rats that were trained extensively on the initial problem. These rats had similar performance and neural activity during prior training and the main effects reported below were the same without their inclusion (Extended Data Fig. 5). One group (*n* = 3) received light to inactivate the ventral subiculum during learning of both new problems, whereas the other (*n* = 3) received ineffective light to serve as controls. Learning and changes in neural correlates on the two problems were similar, thus we collapsed them for our analysis.

**Fig. 6:**
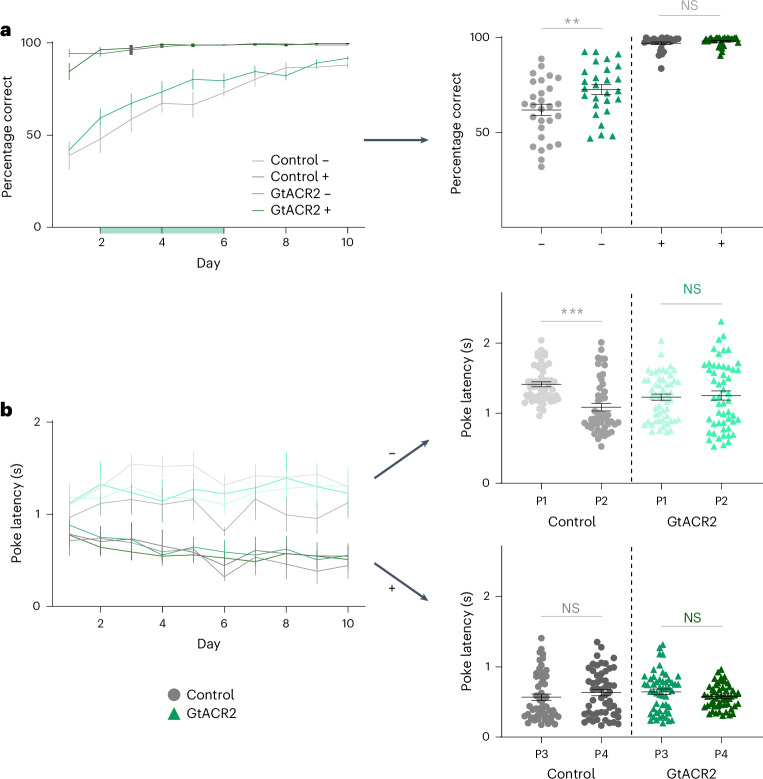
Ventral subiculum inactivation affects behavior during learning of a new problem. **a**,**b**, Percentage correct (**a**) and trial initiation latencies (**b**) across days of learning for rats in the control and GtACR2 groups. The ANOVAs revealed significant effects of session, trial type, group, an interaction between session and trial type, and an interaction between trial type and group (*F* > 5.4; *P* < 0.021; ${{{\eta }}}_{{\rm{p}}}^{2}$ > 0.03; *n* = 10 d for both control and GtACR2) in the percentage correct, reflecting quicker development of the no-go response on nonrewarded positions in the inactivated group at the early stages of learning (days 2–6) (− in scatter plots: *t*_53_ = 2.7; *P* = 9.4 × 10^−3^; two-tailed Student’s *t*-test; *n* = 28 for control; *n* = 27 for GtACR2) and a significant main effect of trial type and an interaction between group and trial type (*F* > 5.8, *P* < 0.0007, ${{{\eta }}}_{{\rm{p}}}^{2}$ > 0.043; *n* = 10 d for both control and GtACR2) in the trial initiation latencies, reflecting a failure of rats in the inactivated group to distinguish the two nonrewarded positions (P1 versus P2 in the scatter plots) (control: *t*_104_ = 5.2, *P* = 1.2 × 10^−6^; *n* = 53 sessions; GtACR2: *t*_104_ = 0.29, *P* = 0.77; two-tailed Student’s *t*-test; *n* = 53 sessions). −, nonrewarded trials; +, rewarded trials. The error bars are the s.e.m. (***P < 0.001; **P < 0.01).

The performance of rats in both groups initially declined when new odors were presented, with rats in both groups often responding to nonrewarded odors (Fig. 6a) and showing a loss of latency effects that required attention to the sequences (Fig. 6b). This decline is consistent with the proposal that they were using the odor sequences (rather than a pattern of motor behaviors or something else that had not changed) to guide their behavior. However, after this initial decline, both groups quickly learned to discriminate rewarded positions accurately (Fig. 6a) and to show differences in their trial initiation latencies reflective of the odor sequences (Fig. 6b). These behaviors developed within the first few sessions, which was quicker than the weeks of training required on the initial problem (before recording). This is consistent with the development of a schema for learning the basic odor discriminations and the sequences in which the odors were embedded. However, there were modest differences between the two groups during acquisition of these new problems, which were not evident on the established problem. Inactivated rats were faster to stop responding at norewarded positions (Fig. 6a; scatters, nonrewarded (−)) and failed to distinguish between these positions in their trial initiation speeds (Fig. 6b; scatters, P1 versus P2).

Against this backdrop, we recorded single units from each group on each day of training (*n* = 79–209 per group per d). Neurons generally exhibited a similar pattern of modulation by epoch as they had on the established problem, although there were some small differences between the two groups (Supplementary Fig. 7).

**Fig. 7:**
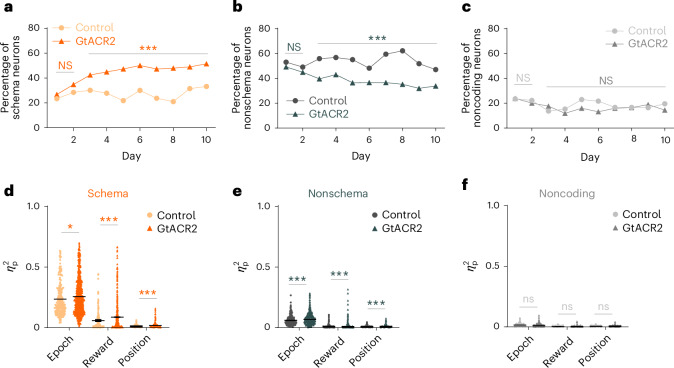
Ventral subiculum inactivation facilitates the formation of schema cells in the OFC during learning of a new problem. **a**–**c**, Percentage of cells recorded on each day that met criteria as schema (**a**), nonschema (**b**) and noncoding (**c**) neurons in each group. The prevalence of schema and nonschema neurons in the two groups were similar initially and then diverged thereafter, with schema neurons increasing more rapidly (overall: *χ*^2^ = 78.9, *P* = 6.6 × 10^−19^; days 1–2: *χ*^2^ = 1.6; *P* = 0.21; days 3–10: *χ*^2^ = 83.0, *P* = 8.0 × 10^−20^; d.f. = 1; *χ*^2^ test) and nonschema neurons declining more rapidly (overall: *χ*^2^ = 59.2, *P* = 1.4 × 10^−14^; days 1–2: *χ*^2^ = 0.87; *P* = 0.35; days 3–10: *χ*^2^ = 66.3, *P* = 3.9 × 10^−16^; d.f. = 1; *χ*^2^ test) in the inactivated group, with no effects of learning or inactivation on the noncoding neurons (*χ*^2^ < 1.8; *P* > 0.18; d.f. = 1; *χ*^2^ test). **d**–**f**, Average explained variance for each factor (epoch, reward, position) within the maze in the schema (**d**; *n* = 260 for control; *n* = 863 for GtACR2), nonschema (**e**; *n* = 516 for control; *n* = 758 for GtACR2) and noncoding (**f**; *n* = 181 for control; *n* = 330 for GtACR2) populations. Inactivation resulted in modest but significant increases in all three kinds of information in the schema and nonschema neurons (schema: *P* = 0.036; 1.5 × 10^−4^; 2.6 × 10^−7^; two-tailed Student’s *t-*test; nonschema: *P* = 5.8 × 10^−8^; 2.1 × 10^−5^; 3.6 × 10^−9^; two-tailed Student’s *t*-test; noncoding: *P* = 0.87, 0.40, 0.76, two-tailed Student’s *t*-test). No adjustments were made for multiple comparisons (***P < 0.001; *P < 0.05; Supplementary Figs. 8 and 9).

Using the approach applied above, we again tracked the development of neural correlates related to trial epoch, reward and position and their generalization across the two mazes during learning. This analysis showed that, in both groups, generalized coding across mazes declined significantly on the new problems in day 1 of training from what had been observed on the established problem, whereas the prevalence of nonschema (but not noncoding) cells increased somewhat in both groups (Fig. 7a–c; day 1). From this low, the prevalence of schema cells increased (and that of nonschema cells decreased) with training; however, these changes were significantly greater in rats in which the ventral subiculum was inactivated during each trial, such that, by day 3, the prevalence of schema cells in the inactivated group became significantly higher than that in controls (Fig. 7a). This increase in the prevalence of generalized representations came at the expense of the nonschema cells, which declined more in the inactivated group than in controls (Fig. 7b and Supplementary Fig. 8) and occurred while the information available about epoch, reward and position—measured as explained variance—was higher on average in the inactivated group for neurons in both categories (Fig. 7d–f and Supplementary Fig. 9). Thus, the effect of inactivation on generalized activity across mazes was not dependent on a decline or population-specific change in information represented within each maze.

**Fig. 8:**
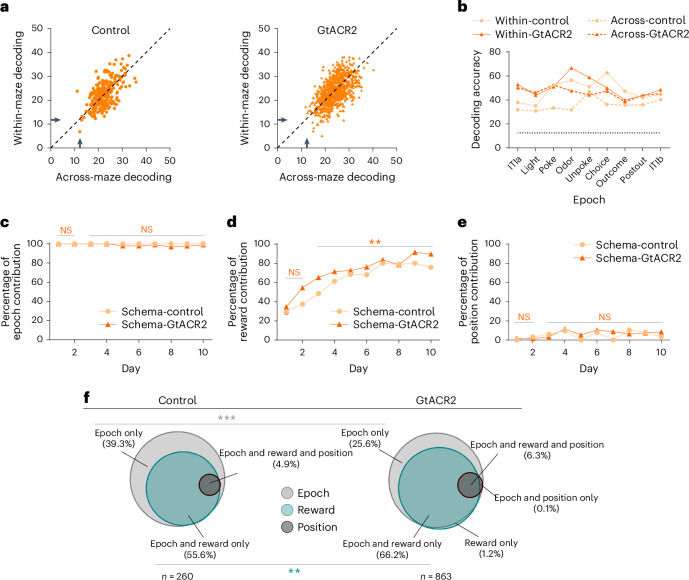
Ventral subiculum inactivation increases the effect of reward on schema cell formation in the OFC during learning of a new problem. **a**, Accuracy of decoding position across all epochs by individual schema cells, where → denotes chance decoding of 12.5%. One-way ANOVA showed that accuracy was similar for decoding within and across mazes for neurons in control (*F*_(1,518)_ = 0.12, *P* = 0.73, ${{{\eta }}}_{{\rm{p}}}^{2}$ = 2.3 × 10^−4^) and GtACR2 rats (*F*_(1,724)_ = 3.0, *P* = 0.083, ${{{\eta }}}_{{\rm{p}}}^{2}$ = 0.0017), whereas inactivation increased accuracy of decoding (within: *F*_(1,1121)_ = 12.48, *P* = 4.0 × 10^−4^, ${{{\eta }}}_{{\rm{p}}}^{2}$ = 0.011; across: *F*_(1,1121)_ = 36.9, *P* = 1.7 × 10^−9^, ${{{\eta }}}_{{\rm{p}}}^{2}$ = 0.032). **b**, Accuracy of decoding position within each epoch by ensembles of schema cells, where dotted line denotes chance decoding of 12.5%. One-way ANOVA showed thta accuracy was greater within than across mazes for neurons in control (*F*_(1,16)_ = 12.1, *P* = 3.1 × 10^−4^, ${{{\eta }}}_{{\rm{p}}}^{2}$ = 0.43) but not GtACR2 rats (*F*_(1,16)_ = 2.1, *P* = 0.17, ${{{\eta }}}_{{\rm{p}}}^{2}$ = 0.11), and inactivation caused better decoding across (*F*_(1,16)_ = 23.4, *P* = 2.0 × 10^−4^, ${{{\eta }}}_{{\rm{p}}}^{2}$ = 0.59) but not within the maze (*F*_*(*__1,16)_ = 0.52, *P* = 0.48, ${{{\eta }}}_{{\rm{p}}}^{2}$ = 0.032). **c**–**e**, Percentage of schema neurons with correlated activity across mazes affected by shuffling (as in Fig. 5a–c) to disrupt information related to epoch (**c**), reward (**d**) or position (**e**). No significant differences between the two groups were observed for either epoch or position (*χ*^2^ < 3.3, *P* > 0.067, d.f. = 1; *χ*^2^ test), whereas the influence of reward grew modestly but significantly faster with inactivation (overall schema: *χ*^2^ = 17.1, *P* = 3.6 × 10^−5^; days 1–2: *χ*^2^ = 2.2, *P* = 0.14; days 3–10: *χ*^2^ = 8.9, *P* = 0.0028; d.f. = 1; *χ*^2^ test). **f**, Venn diagrams summarizing data from **c**–**e**, showing the fraction of schema neurons recorded in control and GtACR2 sessions that were affected by shuffling of information related to epoch (light gray), reward (light green) and position (dark gray) as in Fig. 5d. Sizes of circles are normalized to the total number of neurons recorded in each group, averaged across days (see Extended Data Fig. 6 for the same illustration by day). The proportions in each category and overlap between categories were affected by inactivation, with an increase in those affected by epoch and reward (*χ*^2^ = 9.8; *P* = 0.0018; d.f. = 1; *χ*^2^ test) and a corresponding decrease in those affected by epoch only (*χ*^2^ = 18.4; *P* = 1.8 × 10^−5^; d.f. = 1; *χ*^2^ test). The error bars are the s.e.m. (***P < 0.001, **P < 0.01; Extended Data Figs. 6 and 7 and Supplementary Figs. 10–12).

Inactivation also increased the strength of generalized activity within the schema population. To show this, we again used activity at each position in one maze as the training set for classification of trials drawn at random from either that maze or the other maze on which that neuron was characterized in a particular session. Classification performance was higher across mazes for both individual schema cells (Fig. 8a), as well as for ensembles composed of schema cells (Fig. 8b) when the ventral subiculum was inactivated. Moreover, the classification performance of schema cells surpassed that of both nonschema and noncoding cells. This superiority was observed not only for individual cells (Supplementary Fig. 10) but also for ensembles (Supplementary Fig. 11).

**Extended Data Fig. 6:**
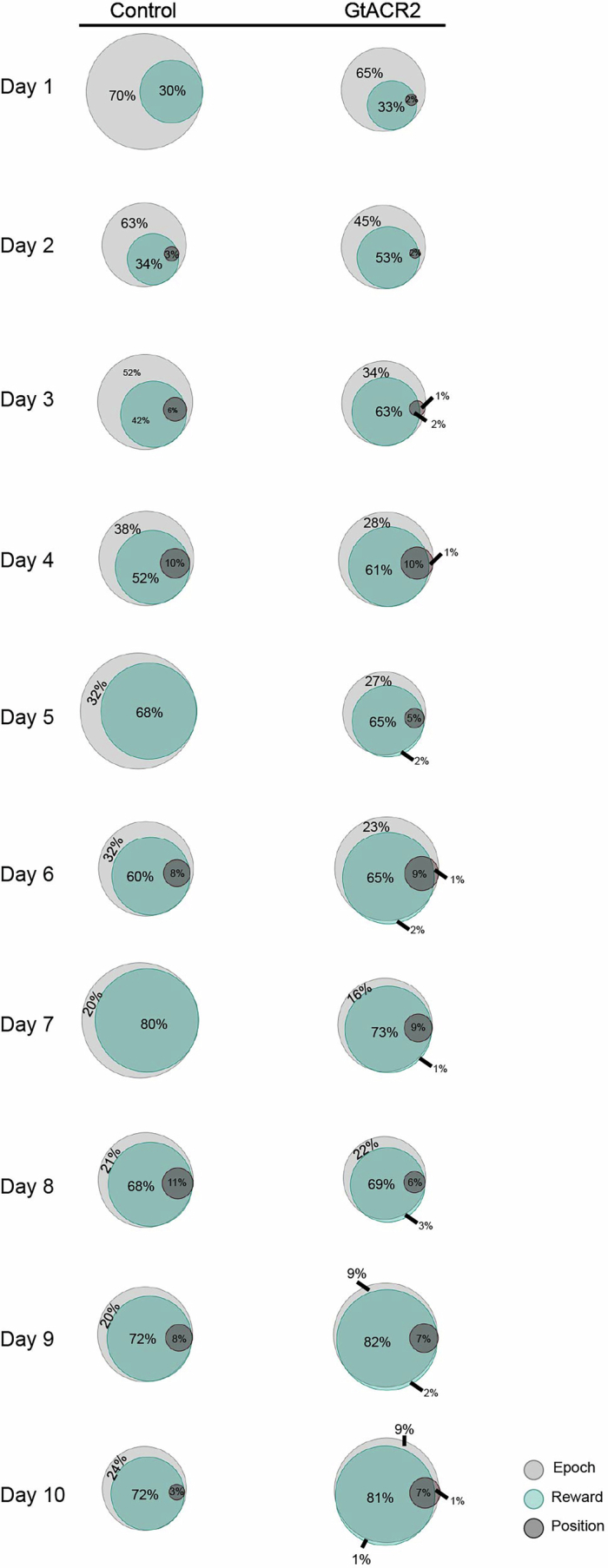
Ventral subiculum inactivation facilitates the formation of schema cells in OFC and increases the effect of reward during learning of a new problem. Venn diagrams show the percentage of schema neurons that were recorded during control and GtACR2 sessions and how they were influenced by information related to epoch (light gray), reward (light green), and position (dark gray) across days. Size of figures reflect overall proportion of neurons that categorized as schema cells, which increased more rapidly in the inactivated group, with an expansion of the reward group and its overlap with epoch and position.

**Extended Data Fig. 7:**
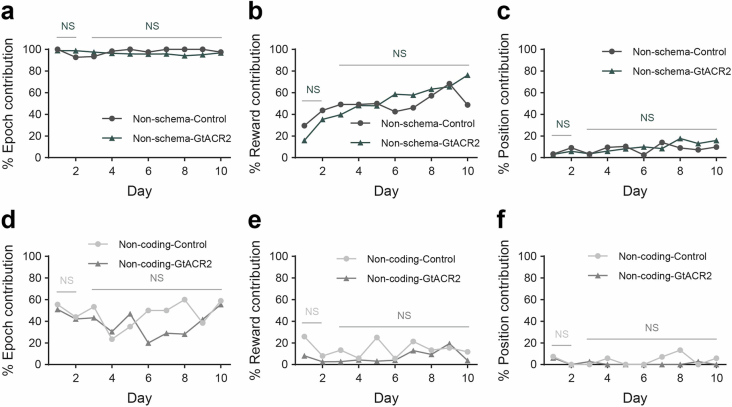
Ventral subiculum inactivation does not affect the influence of epoch, reward, or position on correlated firing in the non-schema and non-coding populations during learning of a new problem. **a-c**, Percentage of non-schema neurons on each day of learning, whose correlated activity across mazes was affected by shuffling to disrupt information related to epoch (**a**), reward (**b**), or position (**c**). **d-f**, Percentage of non-coding neurons on each day of learning, whose correlated activity across mazes was affected by shuffling to disrupt information related to epoch (**d**), reward (**e**), or position (**f**). No significant differences between the two groups were observed (χ^2^< 4.8; P > 0.028; d.f. = 1; χ^2^ test). (NS, not significant).

Finally, although inactivation greatly facilitated the apparent transition from nongeneralized to generalized representations in the OFC (Fig. 7a,b), it had this effect without dramatically disrupting the influence of epoch, reward or positional information on this correlated firing, as revealed by shuffling information within each of these dimensions. The influence of the trial epoch remained high throughout training and was not impacted by inactivation (Fig. 8c,f). The influence of reward and positional information declined in both groups when new problems were introduced (Fig. 8d–f) and then increased with training, with a significant effect of inactivation evident only on the influence of reward (Fig. 8d,f and Extended Data Fig. 6). These changes were again similar when the analysis was restricted to the early periods of the trial (ITIa → odor), which were uncontaminated by external differences related to the go or no-go responses or the presence of reward (Supplementary Fig. 12), and there was no impact of inactivation on the influence of these variables on the degree of correlated activity across mazes in the neurons not classified as schema cells (Extended Data Fig. 7).

**Extended Data Fig. 8:**
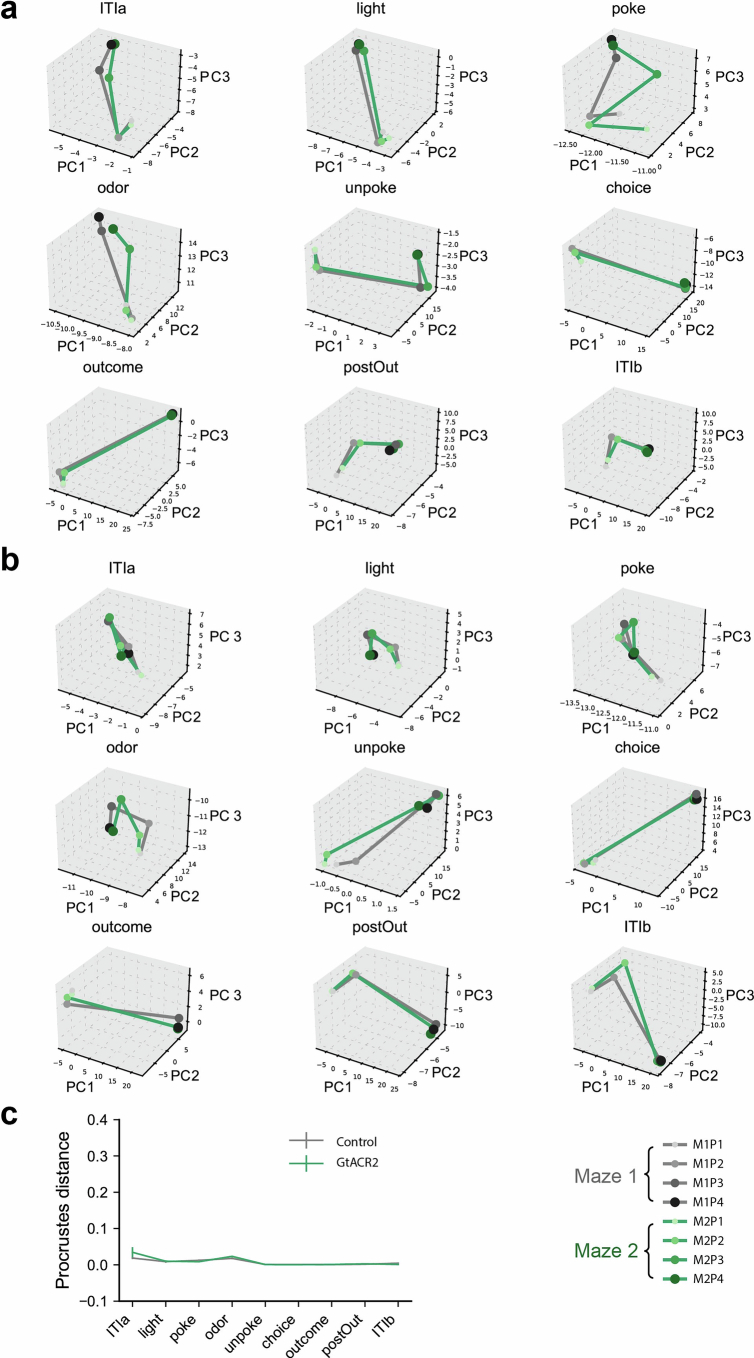
Geometric similarity of task representations across mazes for well-learned task. The positions in principal component space for each maze are shown for both the Control (**a**) and GtACR2 (**b**) groups during each epoch of the well-learned task. For each epoch, gray lines represent Maze 1, while green lines correspond to Maze 2. The four dots on each line, which signify points P1, P2, P3, and P4, transition in color from light to dark in this order. Additionally, the marker size increases progressively from P1 to P4. P1 and P2 represent the common arms that share the same odor, while P3 and P4 correspond to the unique arms with distinct odors. **c**, Quantification of Procrustes analysis for the Control and GtACR2 groups in (**a**) and (**b**) was used to measure the geometric similarity between Maze 1 and Maze 2. No significant difference in geometric similarity between Maze 1 and Maze 2 was observed between the two groups across epochs (t_35_ = 1.1, P = 0.28; two-tailed Student’s t-test; n = 36 for Control and GtACR2). Error bars are SEM.

**Extended Data Fig. 9:**
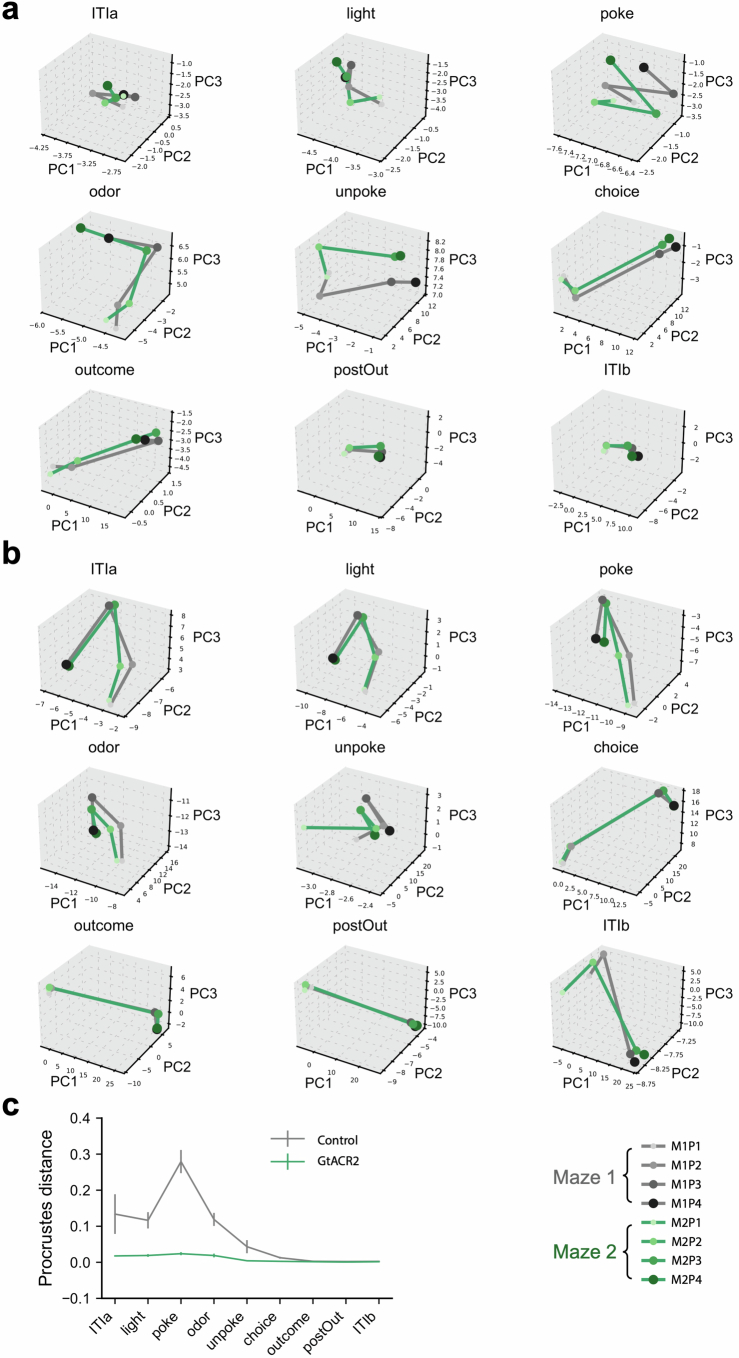
Geometric similarity of task representations across mazes for learning of new task. Geometric similarity of task representations across mazes during new task learning. The positions in principal component space for each maze are shown for the Control (**a**) and GtACR2 groups (**b**) at each learning epoch. Gray lines represent Maze 1, and green lines represent Maze 2. In each epoch, four dots on each line, corresponding to positions P1, P2, P3, and P4, transition from light to dark, with marker sizes increasing progressively from P1 to P4. P1 and P2 represent the common arms, both sharing the same odor, while P3 and P4 correspond to the unique arms, each with distinct odors. **c**, Procrustes analysis was performed for the Control and GtACR2 groups in (**a**) and (**b**) to evaluate the geometric similarity between Maze 1 and Maze 2. Across epochs, no significant differences in geometric similarity between the two mazes were observed between the groups (t_35_ = 4.4, P = 1.1 × 10^−4^; two-tailed Student’s t-test; n = 36 for Control and GtACR2). Error bars are SEM.

**Extended Data Fig. 10:**
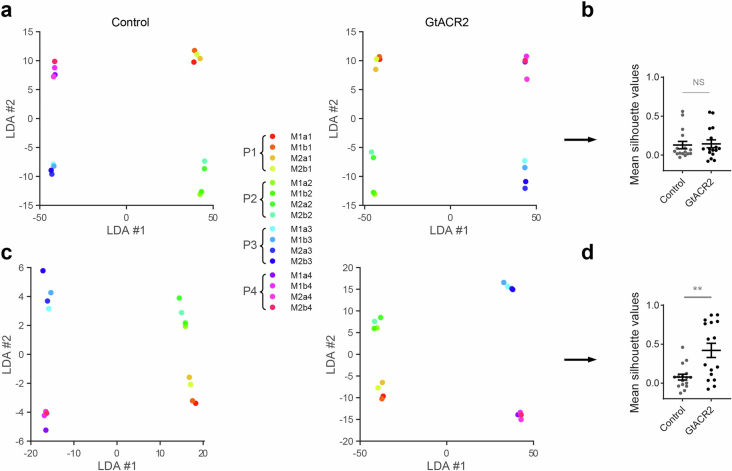
LDA Clustering Plots. Scatter plots of the first two averaged LDAs are shown for both the well-learned task (**a**-**b**) and the learning task (**c**-**d**), combining all sessions. The task includes 16 trial types, each represented by a colored dot. Ten odors were organized into two mazes (Maze 1 and Maze 2), each consisting of two subsequences (**a** and **b**). Each subsequence contains four positions (P1–P4). For example, M1a1 refers to Maze 1, sequence a, position 1. Trials from each position clustered together. The positions are classified as P1, P2, P3, and P4, as indicated in the figure legend. **b**, Quantification of the mean silhouette value for each trial type was performed for both the Control and GtACR2 groups during the well-learned task. No significant difference between the two groups was observed (t_30_ = 0.24, P = 0.81; two-tailed Student’s t-test). **d**, Quantification of the mean silhouette value for each trial type in the learning task showed that the GtACR2 group had significantly higher silhouette values compared to the Control group (t_30_ = 3.5, P = 1.6 × 10^−3^; two-tailed Student’s t-test). Error bars are SEM. (NS, not significant; **P < 0.01).

Finally, we performed several population-level analyses to confirm the significance of the effects observed at the level of single units. For this, we reduced the dimensionality of the population activity and assessed the geometric similarity of task representations across mazes. Both principal component analysis (PCA) and Isomap techniques uncovered a notable discrepancy in the degree of dissimilarity between the control and GtACR2 groups during transfer to new problems (Extended Data Fig. 9 and Supplementary Fig. 14) but not during performance on the established problem (Extended Data Fig. 8 and Supplementary Fig. 13). Similarly, although a linear discriminant analysis (LDA) clustering analysis effectively differentiated among the four positions, distinguishing between maze 1 and maze 2 for corresponding positions posed challenges for the GtACR2 group across multiple days and aggregated sessions during learning (Extended Data Fig. 10 and Supplementary Fig. 16), but not during performance on the established problem (Extended Data Fig. 10 and Supplementary Fig. 15). The addition, analysis of across-trial learning dynamics using both a six-component and an eight-component, non-negative tensor composition analysis (TCA)^41^ revealed distinct patterns during learning in the control group, which displayed prominent trial types and generally flat early components. By contrast, the GtACR2 group exhibited components linked to learned rewards or reward-related aspects, akin to those seen in both groups during performance on the established problem (Supplementary Figs. 17 and 18). Thus, in each population-level analysis, we recapitulated the results of the single-unit approaches, showing significant effects of inactivation on representations in the OFC during learning of new problems, consistent with the accelerated formation of generalized representations, which were not evident during the performance of an established problem.

## Discussion

With the convergence in hypothesized functions of the OFC and HC, it has become increasingly important to test whether and how they coordinate these functions. Nowhere is this of more interest than in the formation and use of generalized cognitive maps or schemas. In the present study, we show that, at least for a single class of problem, ‘schema cells’ are normally hindered or impeded from developing in the OFC by HC output. Notably, this was not evident in the expression of an existing schema in a known situation; it was apparent only when an existing schema had to be applied or transferred to a new situation—a new problem pair. Under those conditions, OFC neurons recorded in rats with the ventral subiculum inactivated to impair hippocampal output exhibited generalized neural correlates much more quickly and at a much higher prevalence than did neurons recorded in controls, as if hippocampal output were suppressing or interfering with the ability of the OFC to carry the schema to the new problem. Although surprising, this effect accords well with evidence of normal learning set formation and even facilitated reversal learning in rats after lesions affecting hippocampal output in rats^42^. Indeed, even in relatively complex OFC-dependent tasks, such as occasion setting or delayed nonmatching, the HC contribution is often limited or nonexistent after a problem has been acquired^16,43^.

In considering this result, it is important to note that the rats were shaped on the task procedures and an initial problem pair before the start of inactivation. Thus, although the OFC did not require the HC to maintain or transfer schema representations to new problems, it is possible that schema formation in the OFC would depend transiently on the HC during this initial stage of learning, as has been shown in other settings^44^. We also disrupted hippocampal output transiently and at the source rather than lesioning or inactivating a particular subregion within the HC or targeting direct projections to or terminals within the OFC. We took this approach, effective in prior work^45^, because we wished to determine how hippocampal outflow affected OFC processing on the fly, with minimum time for any compensation and regardless of the pathway or other areas involved. Thus, the impact of this sudden, transient disruption of the ventral subiculum output on intermediate areas probably shapes our results. One excellent candidate for this might be disruption of interactions with more medial prefrontal areas, which are implicated in switching between or maintaining more context-specific task maps^32,46–48^, failure of which could contribute to the facilitated generalization observed here.

We also did not attempt to distinguish the contribution of different parts of the HC, nor did we argue that inactivation of the ventral subiculum completely silences this complex set of areas. We would speculate that processing in the dorsal HC is most likely responsible for the interference that we observed, because correlates in the dorsal HC are strongly related to external information^49^, whereas recent results have shown that ventral hippocampal activity is more strongly influenced by rewards^50^. Preserved output via pathways like the fornix may also be important in the shape of our effect, especially if these pathways are informationally or functionally biased. In this regard, our results are best viewed as showing one way—rather than the only way—in which the interaction can go awry.

Our approach also used the chemosensory modality, which may hold a special place for the OFC, particularly for rodents, and the goal in this setting—and the information defining the schema—concerns reward, also a feature of the world in which the OFC is often quite interested^51,52^. Although trivial changes, for instance using auditory cues or even spatial locations instead of odors or using food, secondary reinforcers or even punishment in the place of liquid sucrose reward, seem unlikely to be critical, the finding that the HC suppresses schema cell formation in the OFC during learning may depend on the type of information that must be generalized to create the schema cells. In our task, schema cells reflect the collapse of information about specific features of the positions in the two mazes (that is, the particular odors or sequences of odors) in favor of information about the rules that predict reward (that is, the positions in the sequence and/or the reward pattern). The OFC has long been implicated in tracking the conditions predictive of reward^53–55^ and several studies comparing the encoding in the HC and prefrontal areas, including the OFC, have shown that, although both represent similar information, representations in the OFC are skewed to reflect the biological significance of the information, whereas, in the HC, this influence is much less and instead representations seem to be more attuned to external sensory information^10,11,23–27,56^. One way to view this is that both the OFC and the HC contribute to layers of information relevant to cognitive mapping and schemas, with the HC focusing on external specifics that define task states and even episodes perhaps, whereas the OFC warps the map to reflect latent, hidden or internally defined relevance^10,57,58^. This predicts that we might see the opposite result if the external cues were the same between two problems, but the rules governing their relationship to reward differed. Under those conditions, inactivation of hippocampal outflow might be predicted to prevent the formation of schema cells in the OFC, assuming that there were any, because generalization would then depend on the external sensory information. This would be consistent with evidence that hippocampal damage impairs performance in alternative settings and in disambiguating sequences like those used here^59–61^.

A final aspect of the experiment to consider is that, although the results suggest that the OFC is not subordinate to the HC, it does not comment on the reciprocal relationship. We know that hippocampal activity reflects the influence of many attributes related to reward, hidden states or goals, information that the OFC is typically tasked with identifying^19,20,62–65^. Furthermore, prefrontal areas such as the OFC act to shape neural activity in the HC^66,67^. In settings such as the one used in the present study, we would speculate that the OFC probably influences the HC to compress or generalize where external information differs but internal significance is the same, and to split or distinguish states where external information is the same but internal significance differs^68^.

Overall, our findings—that the HC outflow is not necessary to support established schema cells and may at least, under some conditions, inhibit their emergence during transfer to new problems—argue against the idea of a simple feedforward relationship between the HC and the OFC. Instead, these results strongly favor a model in which the OFC and the HC operate in parallel, and perhaps even somewhat in competition, to extract different features defining cognitive maps and schemas. Within this framework, the OFC is predisposed to form representations that more strongly reflect task relevance and motivational goals, which can be at crosspurposes with the function of hippocampal processing.

## Methods

### Participants

The participants were four female and four male Long–Evans rats (Charles River Laboratories, 160–300 g) aged ~3 months at the start of the experiment. Analyses revealed no significant main effect nor any interactions with gender in any of the main findings reported in the text, thus rats of different genders were collapsed in all reported data. Rats were housed individually on a 12-h light:dark cycle at the National Institute on Drug Abuse Intramural Research Program (NIDA-IRP). They received free access to food and water, except during testing periods, when water was removed from their home cages ~23 h before test session. When testing was conducted on consecutive days, they received at least 10 min of free access to water in their home cages after each test session. All test procedures followed the National Institutes of Health (NIH) guidelines and were approved by the Animal Care and Use Committee at the Intramural Research Program (IRP).

### Surgical procedures

Rats were implanted with drivable bundles of 16 nickel–chromium wires (25-mm diameter; AM Systems) targeting the lateral OFC bilaterally (anteroposterior: 3 mm; mediolateral: 3.2 mm). Each wire bundle was housed in a stainless-steel hypodermic tubing (27-gauge, 0.01625-inch outer diameter, 0.01025-inch inner diameter) and cut with a pair of fine bone-cutting spring scissors (Fine Science Tools, cat. no. 16144-13) to extend 1.5–2.0 mm beyond the end of the cannula inside the brain. The tips of the wires were initially implanted 4.2 mm ventral to the brain surface and subsequently advanced during the retraining period to obtain high-quality stable recordings. During the same surgery, pAAV-CKIIa-stGtACR2-FusionRed^34^ was infused bilaterally in the ventral subiculum (6.5 mm posterior and ±4.5 mm lateral to bregma) and optical fibers (Thorlabs) were positioned over each injection site. Rats were given cefalexin (15 mg kg^−1^) orally twice a day for 2 weeks to prevent infection after surgery. Rats were allowed ~4–5 weeks to recover from surgery and to allow viral expression before they began reminder training as described below. Rb polyclonal against tRFP (Evrogen, cat. no. AB233) and Alexa Fluor-647 AffiniPure F(abʹ)_2_ fragment donkey anti-rabbit immunoglobulin G (H (heavy) + L (light)) were used for detection of FusionRed reporter.

### Food neophobia test

Between recording of the initial and learning problems, the rats were food deprived for 48 h and then underwent testing in a HC-dependent neophobia task to confirm the functional efficacy of GtACR2 at inactivating hippocampal outflow. Consumption was tested across five sessions in which rats were placed into a box individually and presented with similar amounts of a familiar (normal chow) and new food (bacon- or banana-flavored sucrose pellets) for 10 min, while receiving light effective at activating the GtACR2 molecule or ineffective light of a similar power as a control. Food ramekins were situated on either side in front of the rat and the orientation of the two foods was counterbalanced within and across days. Both the box and the ramekins were cleaned with wet hand towels and dried after each rat had been tested to reduce any influence of previous tests. Any food remaining after 10 min was collected and weighed to determine consumption.

### Dual figure-of-eight odor-sequence task

Training and recording sessions were conducted in aluminum chambers (~18 inches on a side) outfitted with panels containing an odor port flanked by two fluid-delivery wells, which were monitored by infrared beam sensors across each opening. The odor port was connected to a customized olfactometer, which allowed unique odor cues to be delivered with rapid onset and in a controlled fashion, and the right well allowed delivery of a sucrose reward, all of which was monitored and controlled by customized behavioral software written in C++. Each trial began with illumination of two house lights located above the odor port, which signaled the availability of an odor cue at the port. A stable nosepoke (500 ms) at the odor port initiated odor delivery (500 ms), after which the rats were free to withdraw from the odor port and make a ‘go’ response at the right fluid well. A response on positive trials was considered correct and led to the delivery of 90 μl of a 5% sucrose solution after a random delay (400–1,500 ms) and extinction of the house lights on well exit to start the ITI. A response on negative trials was considered an error and led to immediate extinction of the house lights. If no response was made, which generally occurred only on negative trials where it was correct, the house lights were extinguished after a 2-s period and the trial was considered a ‘no-go’. The ITI period was 4 s after correct trials and 6 s after errors, beginning when the house lights went off.

One of ten odors was delivered to the odor port on each trial and the ten odors were organized into two fixed sequences, the pattern of which constituted what we refer to as nonspatial figure-of-eight mazes (maze 1 and maze 2). Each maze can be broken down into two subsequences as illustrated below, with numbers indicating the unique odor cue and positive (+) and negative (−) symbols to indicate reward availability:

Maze 1:

S1a: 0−, 1−, 2+, 2+;

S1b: 0−, 1−, 3+, 4+;

Maze 2:

S2a: 5−, 6−, 7+, 7+;

S2b: 5−, 6−, 8+, 9+.

Each daily training session consisted of 320 trials, divided into 4 blocks of 80 trials involving 1 of the 2 mazes. Blocks were presented randomly in one of the two orders: maze 1, maze 2, maze 1, maze 2 or maze 2, maze 1, maze 2, maze 1. Before recording, rats were shaped to nosepoke at the odor port and then respond at the well for a reward, after which they were immediately introduced to the odor sequences. All rats (*n* = 8) were trained until they were able to reliably complete the 320 trials each day at a criterion of >75% correct performance on every position (35–45 sessions), after which electrode arrays were implanted in the OFC. Subsequently, all rats (*n* = 8) received additional reminder training after surgery, after which recording began.

Recording during accurate performance on the initial maze pair was divided into control and inactivation sessions. As described in the optogenetic methods, light with a wavelength effective at activating the GtACR2 molecule was delivered during inactivation sessions, whereas light of a similar power but ineffective wavelength was delivered during control sessions. All rats that participated in this part of the study (*n* = 6) underwent both conditions, with the session type alternating randomly except that the same condition could not occur on 3 d consecutively. This resulted in eight control and eight inactivation sessions from all rats but one, which fell ill after completing only three sessions of each type. Data from these sessions were included but the effects reported do not depend on them. An additional rat from the original six became ill in the weeks following the end of recording and had to be removed from the remainder of the study.

After recording on the initial maze pair, the four rats that remained, along with two new rats, were divided into control and inactivation groups (*n* = 3). The two new rats—one in each group—were required to replace the rats that were removed during previous training. These new rats received shaping, surgery, recovery and additional reminder training on the initial problem, as described above, to parallel the training of the original rats. Critically, the original rats placed in the two groups had exhibited similar proportions of schema cells during the previous training and the new rats that were added exhibited proportions of schema cells during their reminder training like the original rats (Extended Data Fig. 5). Single-unit activity was recorded as the rats in these two groups learned two new maze pairs. Each new maze pair consisted of ten new odor cues, arranged in sequences with the same structure as the initial maze pair. Recording continued for 10–12 d on each new pair and the resultant analyses focused on days 1–9 and the final day of recording.

### Single-unit recording

Plexon OmniPlex systems were used to record electrophysiological signals. Neural signals were digitized, amplified and bandpass filtered (250–8,000 Hz) to isolate spike activity, and a threshold was set manually for each active channel to capture unsorted spikes. Timestamps for behavioral events were sent to the Plexon system, synchronized and recorded alongside the neural activity. Spikes were sorted to remove noise and identify single units offline using Offline Sorter (v.4.0; Plexon) with a built-in template-matching algorithm. Sorted files were sent to NeuroExplorer (Nex Technologies) to extract unit and behavioral event timestamps, which were then exported as MATLAB (2021b; MathWorks) formatted files for further analysis. Electrodes were not advanced within a given problem; however, we make no claims about whether single units recorded on different days within the same problem are the same or different neurons. The electrodes were advanced ~120 μm to change the neural population being sampled between odor problems.

### Optogenetic stimulation

Light was delivered using a combined optogenetic–electrical commutator interfaced with custom-made 1.25-mm FC ferrules (Thorlabs). To inactivate the ventral subiculum, 465-nm light (6–10 mW of power output) was delivered to activate GtACR2 and suppress neural activity. As a control, 630-nm light (6–10 mW of power output) was delivered. This wavelength falls outside the frequency sensitivity range of the GtACR2 molecule^35^. In the figure-of-eight task, light was delivered continuously (that is, not pulsed) during each trial, starting with house light illumination and terminating after outcome delivery. During recording on the initial maze, each rat received both control and inactivating wavelengths of light in different sessions, alternating pseudorandomly. During recording on subsequent mazes, each rat received either control or inactivating light. In the neophobia task, light was delivered continuously for the full 10-min test period and each rat received either control or inactivating light.

### Peri-event time epochs

Each trial was divided into nine epochs associated with different events:

1. ITIa = time from 500 ms before to light onset
2. light = 0 ms before to 500 ms after light onset
3. poke = 0 ms before to 500 ms after odor port nosepoke
4. odor = 0 ms before to 500 ms after odor delivery
5. unpoke = 0 ms before to 500 ms after odor port unpoke
6. choice = 0 ms before to 500 ms after well entry (or trial termination)
7. outcome = 0 ms before to 500 ms after reward delivery (or 500 ms after trial termination)
8. postout = 500 ms after end of outcome period
9. ITIb = 500 ms after end of postout period.

The spike trains for every isolated unit were aligned with the onset of each event. Spike number was counted with a bin = 100 ms. A Gaussian kernel (*s* = 50 ms) was used to smooth the spike train on each trial. The number of rats and neurons were not predetermined by any statistical methods, but are comparable to those reported in previous publications from our and other labs. All data were analyzed using MATLAB (R2021b). The error bars in figures denote the s.e.m.

### Single-neuron selectivity and calculation of explained variance

The firing rate of each neuron was assessed by three-way analysis of variance (ANOVA) to determine whether it was selective to epoch, reward or position (*P* < 0.01). For each epoch-selective neuron, the maximal firing rate at all epochs was found, then the percentage of each epoch was calculated. For each position-selective neuron, the maximal firing rate across all positions (P1, P2, P3 and P4) was found and the respective percentage for each position calculated. For reward-selective neurons, the maximal firing rate at rewarded or nonrewarded trials was assessed and the percentage for each reward category calculated. The partial *η*^2^ for each neuron was computed after a three-way ANOVA to determine the explained variance.

### Calculation of cross-maze correlations and the effect of shuffling information about epoch, reward and position

We performed correlation analyses to compare the activity of each single unit across problems. Each maze comprises 8 positions and, for each position, there are 20 trials. When considering 9 epochs, this configuration results in a 160 × 9 matrix for each maze. The mean firing rate of 8 trial types and 9 epochs resulted in a 72 × 1 matrix for each maze. A unit was categorized as a schema cell if it exhibited a strong correlation for the 72 × 1 matrix for both mazes (corrcoef, MATLAB > 0.8) at *P* < 0.01; it was categorized as a nonschema cell if *r* ≥ 0.4 and *r* ≤ 0.8, although a noncoding cell would be the unit with a correlation <0.4. Subsequently, the influence of epoch, reward and position on the correlation was determined by shuffling information for each maze separately using all trials with a dimension of 160 × 9 matrix 1,000× and comparing the distribution of shuffled correlations with the original correlation coefficient for a given unit (*r*1). If *r*1 > 99% of the shuffled correlation coefficients, we considered the shuffled dimension to have contributed significantly to that unit cross-maze correlation.

1. To determine the influence of epoch, we shuffled activity between epochs within each trial in the 160 × 9 matrix from each maze. This manipulation altered any relationship between firing activity and epoch. while keeping any relationship to reward or position the same.
2. To determine the influence of reward, we shuffled activity between reward categories within each epoch and maze subsequence in the 160 × 9 matrix from each maze. This manipulation altered any relationship between firing activity and reward, while keeping any relationship to epoch or position within subsequence the same.
3. To determine the influence of position (independent of reward), we shuffled activity between positions within each reward category and epoch in the 160 × 9 matrix from each maze. This manipulation altered any relationship between firing activity and position, while keeping any relationship to epoch or reward the same.

### Single-cell and population decoding of position

All 20 trials for each trial type were included for 9 epochs, resulting in a 160 (no. of trials) × 9 (no. of epochs) matrix. Then, randomly one trial of each 8 trial types was left out to get an 8 × 9 test set from maze 1 (within maze), whereas the remaining 152 × 9 matrix was used to train the model. The same size of matrix (8 × 9) from maze 2, with an identical trial index as the test set from maze 1, was used for cross-maze decoding of trial types. Based on the matrix of firing rate, a linear multiclass classification (LIBLINEAR^69^, https://www.csie.ntu.edu.tw/~cjlin/liblinear) was trained to classify eight trial types. This procedure was repeated 1,000×, then the average decoding accuracies within the maze and across the maze were calculated for each cell. The chance level was one in eight for each cell.

For population decoding of position within and across maze for schema cells (*n* = 250), we employed a support vector machine using MATLAB toolboxes libsvm-3.22 and ndt.1.0.4 for binary classification^70,71^. Classification accuracy was evaluated through a leave-one-out crossvalidation procedure. On each repeat, 15 trials for each trial type were randomly selected and included for 9 epochs, resulting in a 120 (no. of trials) × 9 (no. of epochs) matrix for each maze. One trial from each trial type within maze 1 was left out for future testing. Simultaneously, the trial from maze 2, with the corresponding index as the left-out trial in maze 1, was set aside for across maze testing. The classifier was then constructed using the remaining trials from maze 1. The mean decoding accuracy for each trial type of each epoch was computed as the average across 200 repeats.

### Similarity between neuronal responses

We averaged the neuronal responses for each epoch within a trial (nine epochs per trial), resulting in a matrix of size *n*Neuron-by-*n*Trials × *n*Epochs, where *n*Neuron, *n*Trials and *n*Epochs represent the number of collected neurons, trials and epochs, respectively. Subsequently, we applied dimensionality reduction methods to reduce the first neuronal dimension to three components. The neuronal responses from each maze configuration were then projected on to this subspace. To compare the neuronal patterns across different mazes, we performed the Procrustes analysis. This method aligned the neuronal response data by minimizing the Procrustes distance through translation, uniform scaling and rotation of the datasets. Finally, the Procrustes distance, defined as the sum of squared distances between corresponding points in each aligned shape, was computed to quantify dissimilarity between the neuronal responses from different mazes. A smaller Procrustes distance indicates greater similarity between the neuronal responses in two maze configurations.

To enhance the robustness of our findings, we employed a linear and a nonlinear dimension reduction method. PCA, a linear method, was used to reduce the dimensionality by identifying the PCs that capture the most variance in the data. The first three components capture percentage of variance. In addition, we applied Isomap, a nonlinear method that preserves the geodesic distances between points and maps the intrinsic geometric structure of the data on to a low-dimensional space. The number of neighbors for each point was set to 19 because each condition was repeated 20×.

### Clustering

Data from each experimental group were initially organized into a three-dimensional *K* × *N* × T array, representing the firing rates of *N* neurons across *K* experimental trials for *T* epochs. Subsequently, this array was reshaped into a two-dimensional *K* × (*N* × T) format to facilitate PCA on the combined dataset, yielding PC scores. Using the MATLAB function ‘manova1’, multivariate analysis of variance (MANOVA) was performed on the first 30 PC scores, utilizing the provided trial labels as grouping variables. Canonical variables were extracted from the MANOVA statistics. Furthermore, for each trial type, the mean canonical discriminant scores were calculated. The resultant scores were then utilized to create scatter plots representing the first two LDA dimensions. To evaluate the effectiveness of clustering between the control and GtACR2 groups, mean silhouette value for each trial type in both groups was calculated. The silhouette value measures how closely an object is associated with its own cluster (cohesion) compared with its association with other clusters (separation).

### TCA analysis

For conducting TCA, the firing rates for each group were organized into a three-dimensional array (*N* × *T* × *K*). This array format, often referred to as a third-order tensor (citation), encapsulates the firing activity across multiple trials. After exporting the data as a MATLAB.mat file, we imported it into Spyder, leveraging the TensorTools package developed by Williams et al.^41^, for analysis.

### Statistics and reproducibility

Statistical analyses are detailed in the main text, figure legends and supplementary figure legends. The error bars and shading represent 95% confidence intervals (CIs) unless otherwise specified. For each experiment, the sample size (*n*) and its definition are explicitly provided in the corresponding figure and supplementary figure legends. Statistical significance is indicated as follows: *P < 0.05, **P < 0.01, ***P < 0.001. Pearson’s correlation coefficient is denoted as ‘*r*’. For shuffling analysis, we used a bootstrapping procedure to estimate CIs. This process was repeated 1,000×, with each iteration involving random subsampling of the data. The 1st and 99th percentiles of the resulting distributions were used as CIs. Sample sizes were not predetermined using statistical methods. For the analysis of selected sample sizes, 12 different numbers of selected neurons were chosen to ensure consistency and reproducibility, as shown in Supplementary Figs. 5 and 11. We applied a variety of statistical tests, including nonparametric tests (Wilcoxon’s rank-sum test, Student’s *t*-test, *χ*^2^ test, correlation coefficient test, one-way ANOVA, two-way ANOVA, three-way ANOVA) in MATLAB (v.2021b), as well as one-way ANOVA, two-way ANOVA and unpaired Student’s *t*-tests in GraphPad Prism 10. To account for unequal sample sizes when comparing preferred versus nonpreferred signals, we drew equal-sized bootstrapped data samples and calculated a bootstrap statistic for each trial type using MATLAB’s ‘bootstrp’ function. Statistical significance was assumed for *P* < 0.05. No data were excluded from the analyses and the experiments were randomized. Investigators were blinded to allocation during experiments and all analyses were performed with blinding of the experimental conditions.

### Reporting summary

Further information on research design is available in the Nature Portfolio Reporting Summary linked to this article.

## Online content

Any methods, additional references, Nature Portfolio reporting summaries, source data, extended data, supplementary information, acknowledgements, peer review information; details of author contributions and competing interests; and statements of data and code availability are available at 10.1038/s41593-025-01928-z.

## Supplementary information

Supplementary Information Supplementary Figs. 1–18.

Reporting Summary

## Source data

Source Data Fig. 1 Original confocal image for Fig. 1d.

## Data Availability

The datasets used in the present study are available at https://osf.io/78gy4. Source data are provided with this paper.

## Code availability

All scripts used in the present study are available at https://osf.io/78gy4.
