# Supplementary material for: Hippocampal output suppresses orbitofrontal cortex schema cell formation
Source: Nat Neurosci. 2025 Apr 14;28(5):1048–60. doi: 10.1038/s41593-025-01928-z (PMC12081290; doi:10.1038/s41593-025-01928-z)
Supplement: Supplementary file 1 — Supplementary Figs. 1–18. [file 41593_2025_1928_MOESM1_ESM.pdf]

nature neuroscience

Supplementary information https://doi.org/10.1038/s41593-025-01928-z

Hippocampal output suppresses orbitofrontal cortex schema cell formation

In the format provided by the authors and unedited

Location 1	Location 2	Location 3	Location 4	Location 5	Location 6	Location 7	Location 8
Control	Inactivation	Inactivation	Control	Inactivation	Control	Control	Inactivation
Inactivation	Retraining	Retraining	Inactivation	Retraining	Inactivation	Inactivation	Retraining
Retraining	Control	Control	Retraining	Control	Retraining	Retraining	Control

Supplementary Fig. 1 | Illustration of pseudorandomized ordering of control, inactivation and retraining sessions during performance on the established problem. Each rat completed three session types – inactivation, control, and reminder - at each location during recording. While retraining always followed the inactivation session, the first session of the three was randomly selected from either control or inactivation. As a result, due to the repetition of this design, both inactivation and control sessions could be preceded by either a control or a retraining session.

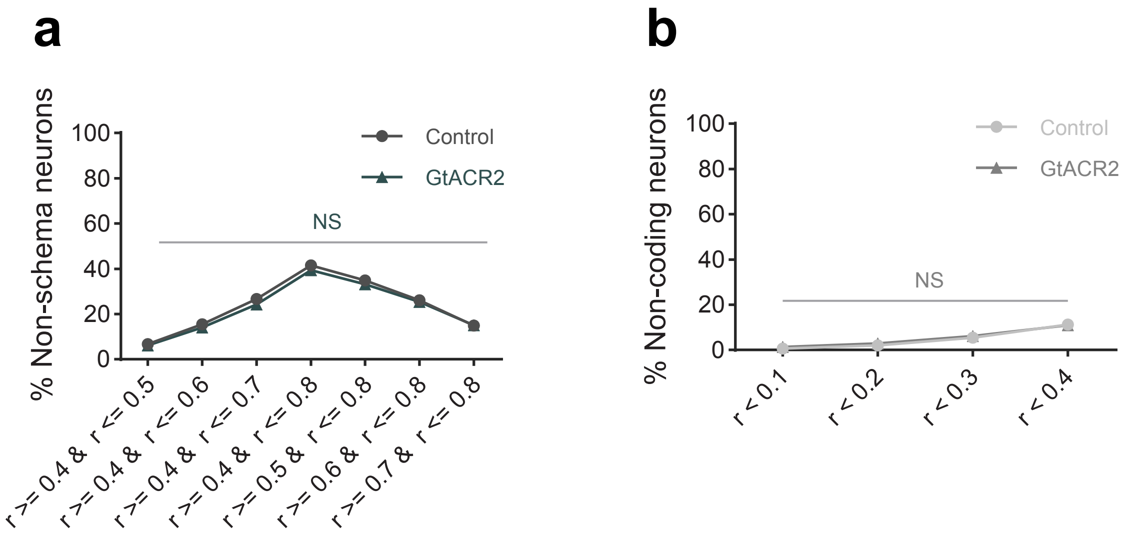
a
% Non-schema neurons
100
80
60
40
20
0
Control
GtACR2
NS
r >= 0.4 & r <= 0.5
r >= 0.4 & r <= 0.6
r >= 0.4 & r <= 0.7
r >= 0.4 & r <= 0.8
r >= 0.5 & r <= 0.8
r >= 0.6 & r <= 0.8
r >= 0.7 & r <= 0.8
b
% Non-coding neurons
100
80
60
40
20
0
Control
GtACR2
NS
r < 0.1
r < 0.2
r < 0.3
r < 0.4

Supplementary Fig. 2 | Various thresholds for non-schema and non-coding neurons for well-learned task. Percentage of non-schema (**a**) and non-coding (**b**) neurons at different thresholds for categorization for well-learned task. No significant difference was observed between the two groups in terms of the proportion of neurons at any threshold value, whether for non-schema or non-coding neurons ($\chi^2 < 5.6$; $P > 0.02$; d.f. = 1; χ^2 test). (NS, not significant).

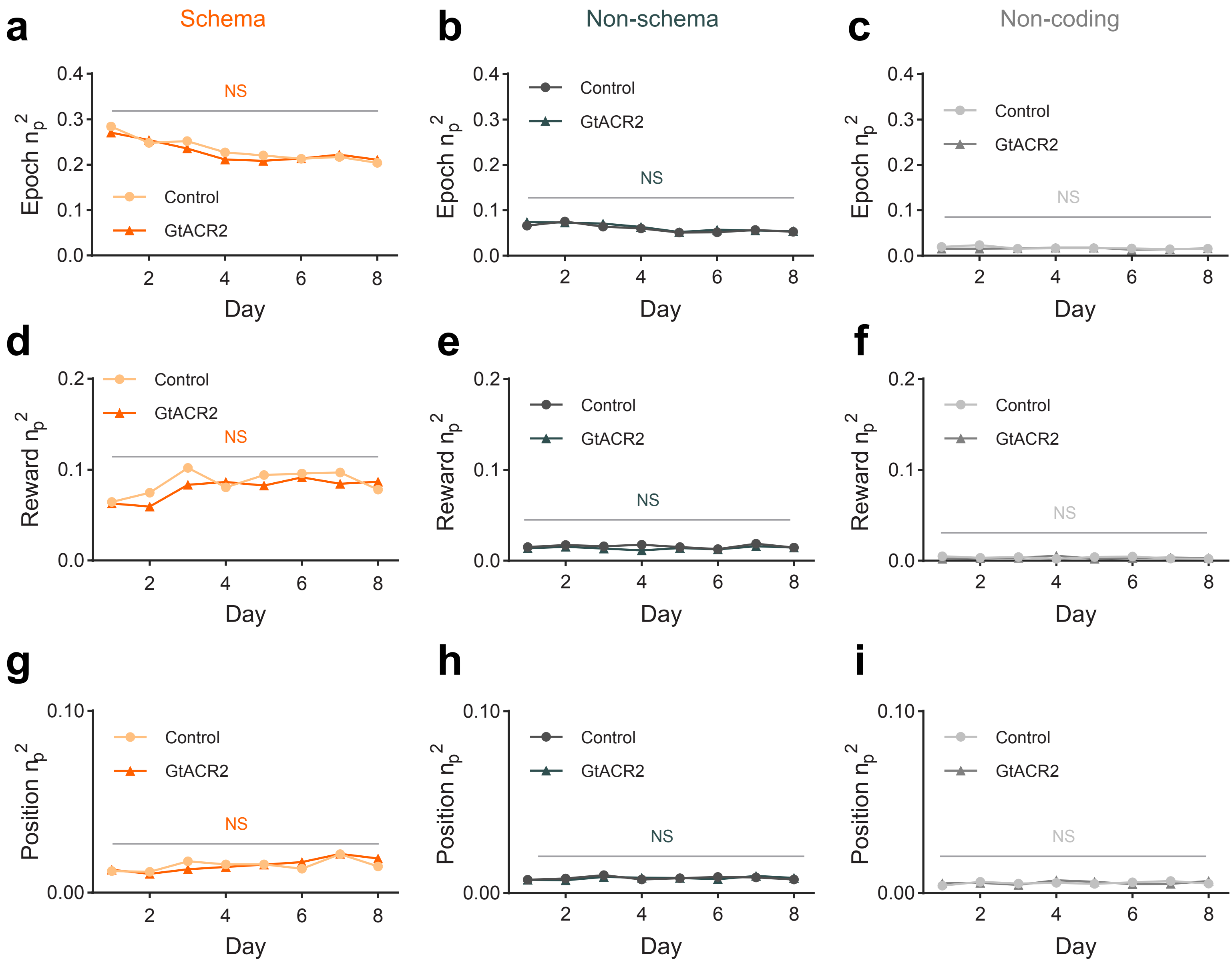

a
Schema
b
Non-schema
c
Non-coding
Epoch n_p^2
Control
GtACR2
NS
Day
d
e
f
Reward n_p^2
g
h
i
Position n_p^2

Supplementary Fig. 3 | Ventral subiculum inactivation does not affect variance explained by epoch, reward, and position during performance on an established problem. Explained variance, averaged across neurons, is plotted for each neural subpopulation (**a**, **d**, **g** - schema, **b**, **e**, **h** - non-schema, and **c**, **f**, **i** - non-coding) on each day of recording; there were no effects of inactivation (schema: $F < 3.0$; $P > 0.13$; non-schema: $F < 7.6$; $P > 0.028$; non-coding: $F < 1.6$; $P > 0.25$; Two-way ANOVA). (NS, not significant).

a

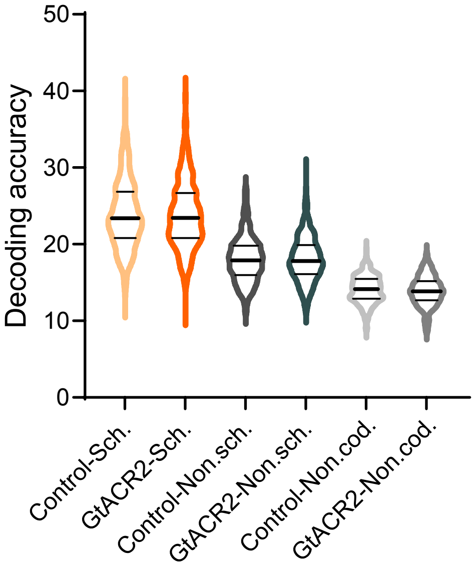
Decoding accuracy
50
40
30
20
10
0
Control-Sch.
GtACR2-Sch.
Control-Non.sch.
GtACR2-Non.sch.
Control-Non.cod.
GtACR2-Non.cod.

Supplementary Fig. 4 | Single-cell decoding performance was evaluated for schema, non-schema, and non-coding neurons in the context of a well-learned task. Mean decoding accuracy across all epochs was assessed for each category of cells(**a**). Significant differences in single-cell decoding were observed among schema, non-schema, and non-coding neurons for either control ($F_{(1,1854)} = 424.2$, $P = 1.0 \times 10^{-15}$, $\eta_p^2 = 0.31$; One-way ANOVA) or GtACR2 groups ($F_{(1,1832)} = 451.0$, $P = 1.0 \times 10^{-15}$, $\eta_p^2 = 0.33$; One-way ANOVA). The chance decoding level is 12.5%.

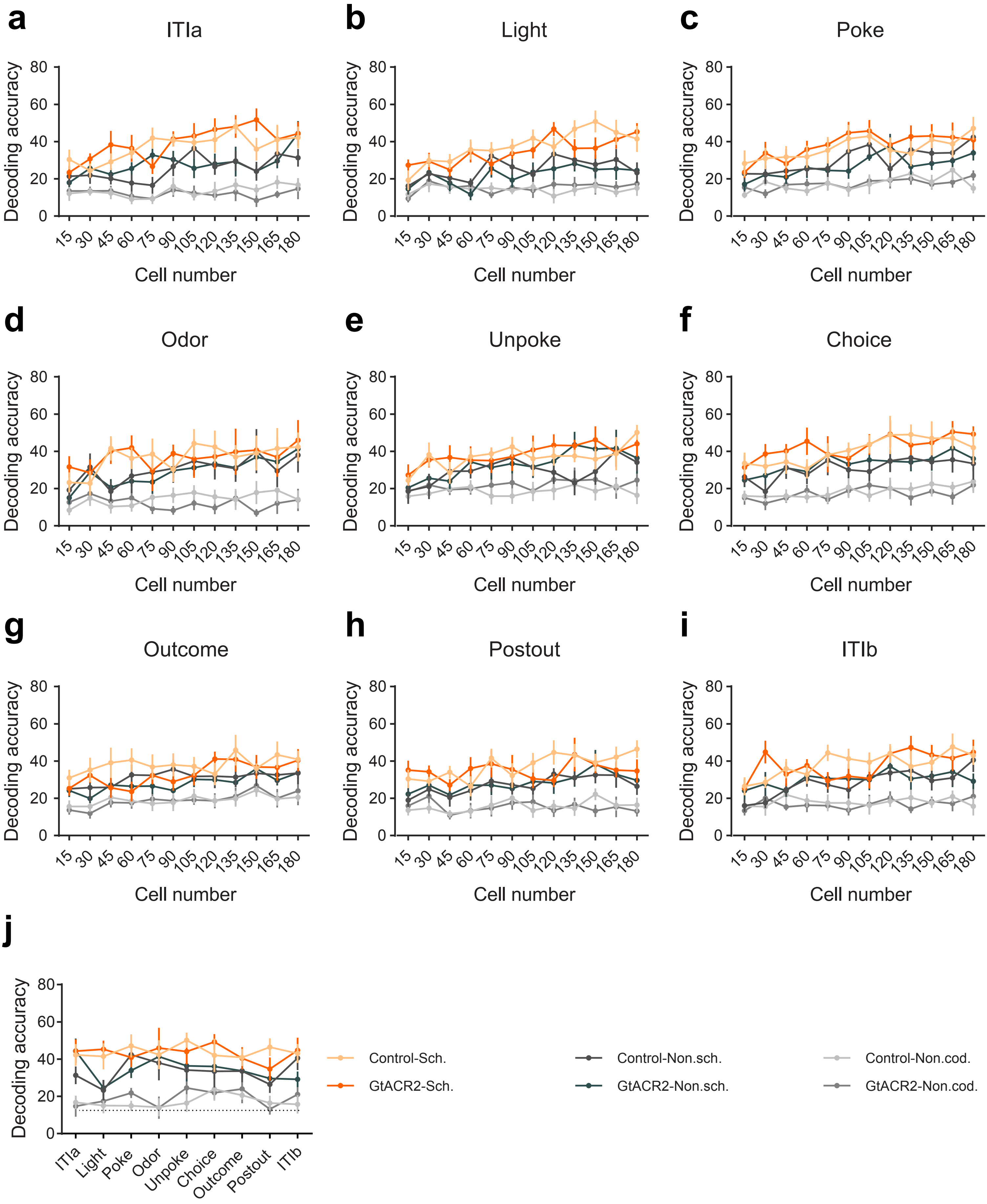

a
ITIa
b
Light
c
Poke
d
Odor
e
Unpoke
f
Choice
g
Outcome
h
Postout
i
ITIb
j
Decoding accuracy
0
20
40
60
80
15
30
45
60
75
90
105
120
135
150
165
180
Cell number
ITIa
Light
Poke
Odor
Unpoke
Choice
Outcome
Postout
ITIb
Control-Sch.
GtACR2-Sch.
Control-Non.sch.
GtACR2-Non.sch.
Control-Non.cod.
GtACR2-Non.cod.

Supplementary Fig. 5 | Population level across maze decoding for well-learned task. The accuracy of decoding across maze positions within each epoch was assessed using ensembles comprising varying numbers of each cell type (**a**-**i**) and a set of 180 cells across epochs (**j**). Significant effects of the three groups (schema, non-schema, and non-coding) were observed for both the Control and GtACR2 groups across each epoch by one-way ANOVA analysis ($P < 1.0 \times 10^{-12}$). Error bars are SEM.

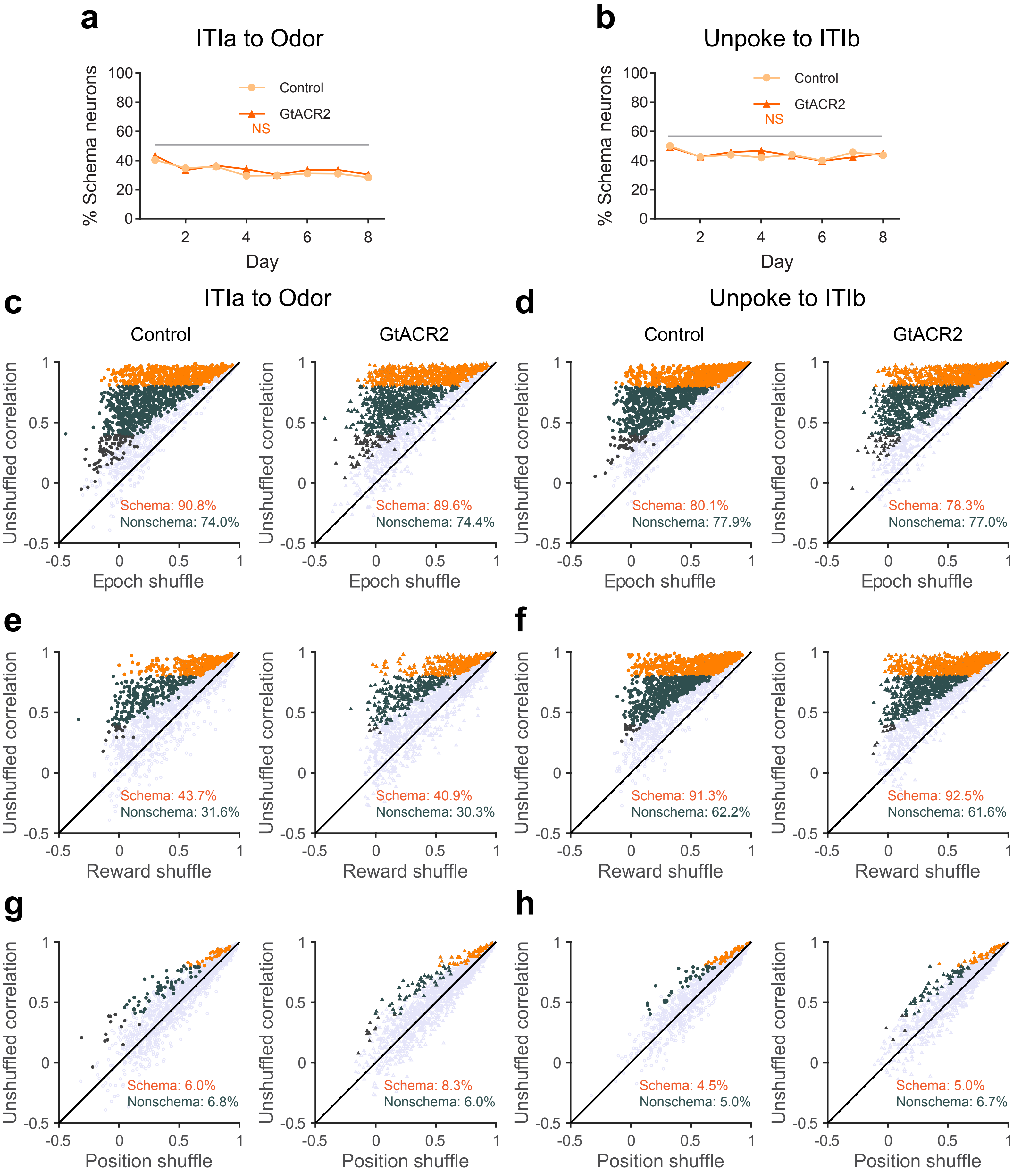

a
ITIa to Odor
% Schema neurons
Control
GtACR2
NS
Day
b
Unpoke to ITIb
% Schema neurons
Control
GtACR2
NS
Day
c
ITIa to Odor
Control
GtACR2
Unshuffled correlation
Epoch shuffle
Schema: 90.8%
Nonschema: 74.0%
Schema: 89.6%
Nonschema: 74.4%
d
Unpoke to ITIb
Control
GtACR2
Schema: 80.1%
Nonschema: 77.9%
Schema: 78.3%
Nonschema: 77.0%
e
Reward shuffle
Schema: 43.7%
Nonschema: 31.6%
Schema: 40.9%
Nonschema: 30.3%
f
Schema: 91.3%
Nonschema: 62.2%
Schema: 92.5%
Nonschema: 61.6%
g
Position shuffle
Schema: 6.0%
Nonschema: 6.8%
Schema: 8.3%
Nonschema: 6.0%
h
Schema: 4.5%
Nonschema: 5.0%
Schema: 5.0%
Nonschema: 6.7%

Supplementary Fig. 6 | Ventral subiculum inactivation does not affect the prevalence or content of schema cells in OFC during performance on an established problem, during either early or late parts of the trial. a. Percentage of schema neurons from control and GtACR2 sessions on each day of training at 0.8 threshold, using only data from ITI->odor, prior to external differences related to responding or reward; there was no difference between the two groups (Overall: $\chi^2 = 1.4$, P = 0.23; d.f. = 1; χ^2 test). **b.** same as panel a, but using data from unpoke->ITIb, where response and reward differed; there was no difference between the two groups (Overall: $\chi^2 = 0.03$, P = 0.86; d.f. = 1; χ^2 test). **c-h**, Scatter plots show the correlation coefficients of each neuron from the control (left panels) and GtACR2 (right panels) sessions. Y-axes plot the correlation coefficients from unshuffled data; the x-axes plot the mean correlation coefficients obtained after shuffling data (1000x) to disrupt contributions of information related to epoch (**c**, **d**), reward (**e**, **f**), or position (**g**, **h**). Orange/gray/black cells had actual correlation coefficients larger than 99% of the shuffled results, indicating a significant contribution of the shuffled type of information to the correlated firing patterns. These populations, the size noted on the panels, were not affected by inactivation either early ($\chi^2 < 4.7$; P > 0.031; d.f. = 1; Chi-squared test) or late ($\chi^2 < 0.84$; P > 0.36; d.f. = 1; Chi-squared test) in the trial. Orange denotes schema cells, and gray represents non-schema cells. NS, not significant.

a

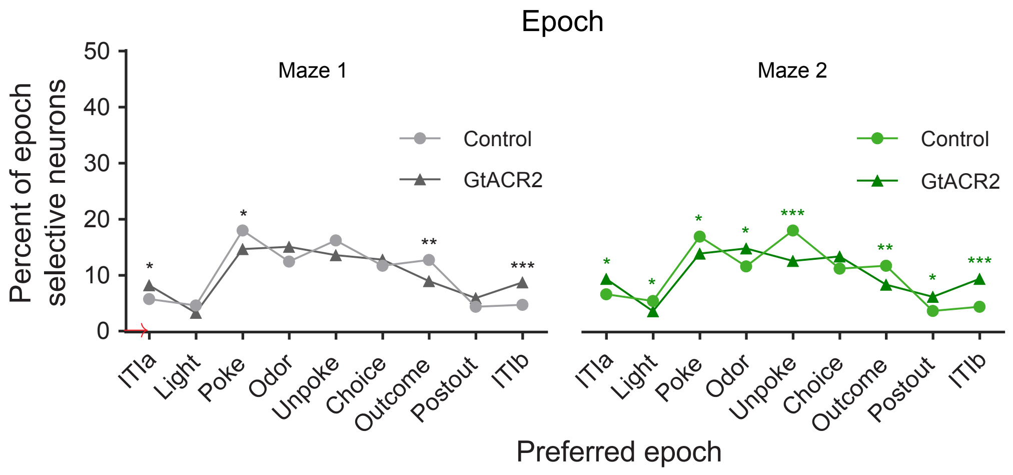

Epoch
Maze 1
Maze 2
Control
GtACR2
Percent of epoch selective neurons
0
10
20
30
40
50
*
*
**
***
*
*
*
*
***
**
*
***
ITIa
Light
Poke
Odor
Unpoke
Choice
Outcome
Postout
ITIb
Preferred epoch

Supplementary Fig. 7 | Quantification of influence of epoch on the learning task. a. Plots show the percentage of the OFC neurons whose firing was significantly modulated by epoch during learning of a new problem (ANOVA, $P < 0.01$), with each neuron assigned to the condition of maximal firing. Significant differences for some epochs were found between Control and GtACR2 groups (Maze 1: P = 0.048; 0.092; 0.047; 0.089; 0.089; 0.42; 0.007; 0.092; 9.2×10^{-4}; d.f. = 1; χ^2 test; Maze 2: P = 0.022; 0.025; 0.034; 0.025; 4.1×10^{-4}; 0.094; 9.6×10^{-3}; 0.011; 2.4×10^{-5}; d.f. = 1; χ^2 test). Red → denotes chance level. False discovery rate (FDR) and Benjamini-Hochberg (BH) corrections were applied to correct for multiple comparisons. (***$P < 0.001$; **$P < 0.01$; *$P < 0.01$).

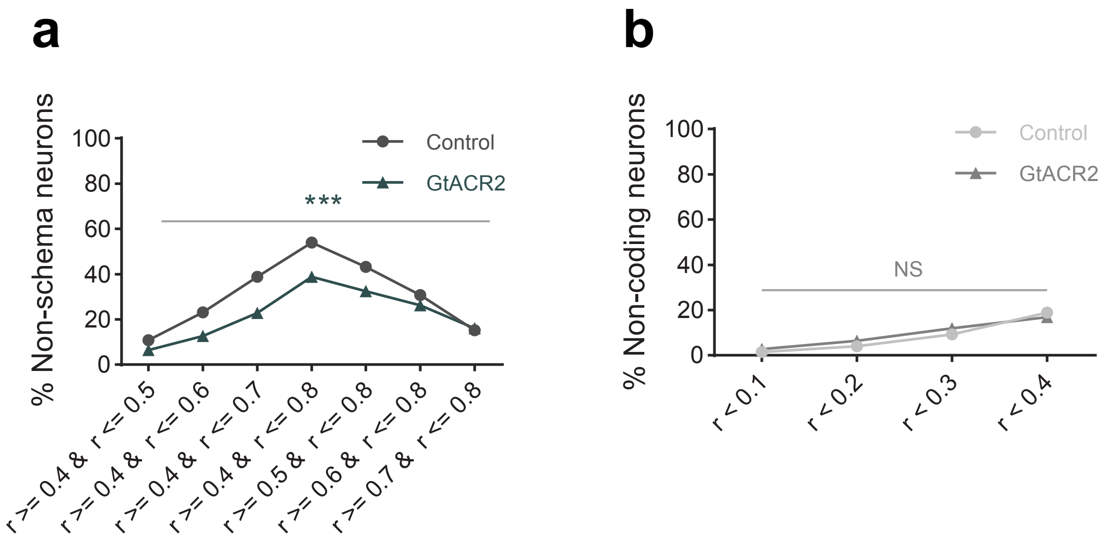

a
% Non-schema neurons
100
80
60
40
20
0
Control
GtACR2
***
r >= 0.4 & r <= 0.5
r >= 0.4 & r <= 0.6
r >= 0.4 & r <= 0.7
r >= 0.4 & r <= 0.8
r >= 0.5 & r <= 0.8
r >= 0.6 & r <= 0.8
r >= 0.7 & r <= 0.8
b
% Non-coding neurons
100
80
60
40
20
0
Control
GtACR2
NS
r < 0.1
r < 0.2
r < 0.3
r < 0.4

Supplementary Fig. 8 | Various thresholds for non-schema and non-coding neurons for learning task. Percentage of non-schema (**a**) and non-coding (**b**) neurons at different thresholds for categorization for learning task. A significant difference was observed between the two groups in terms of the proportion of non-schema neurons using ANOVA ($F_{(1,6)} = 13.8$, $P = 9.9 \times 10^{-3}$, $\eta_p^2 = 0.70$; One-way ANOVA) but not for non-coding neurons at any threshold ($F_{(1,3)} = 1.0$, $P = 0.38$, $\eta_p^2 = 0.25$; One-way ANOVA). (***$P < 0.001$; NS, not significant).

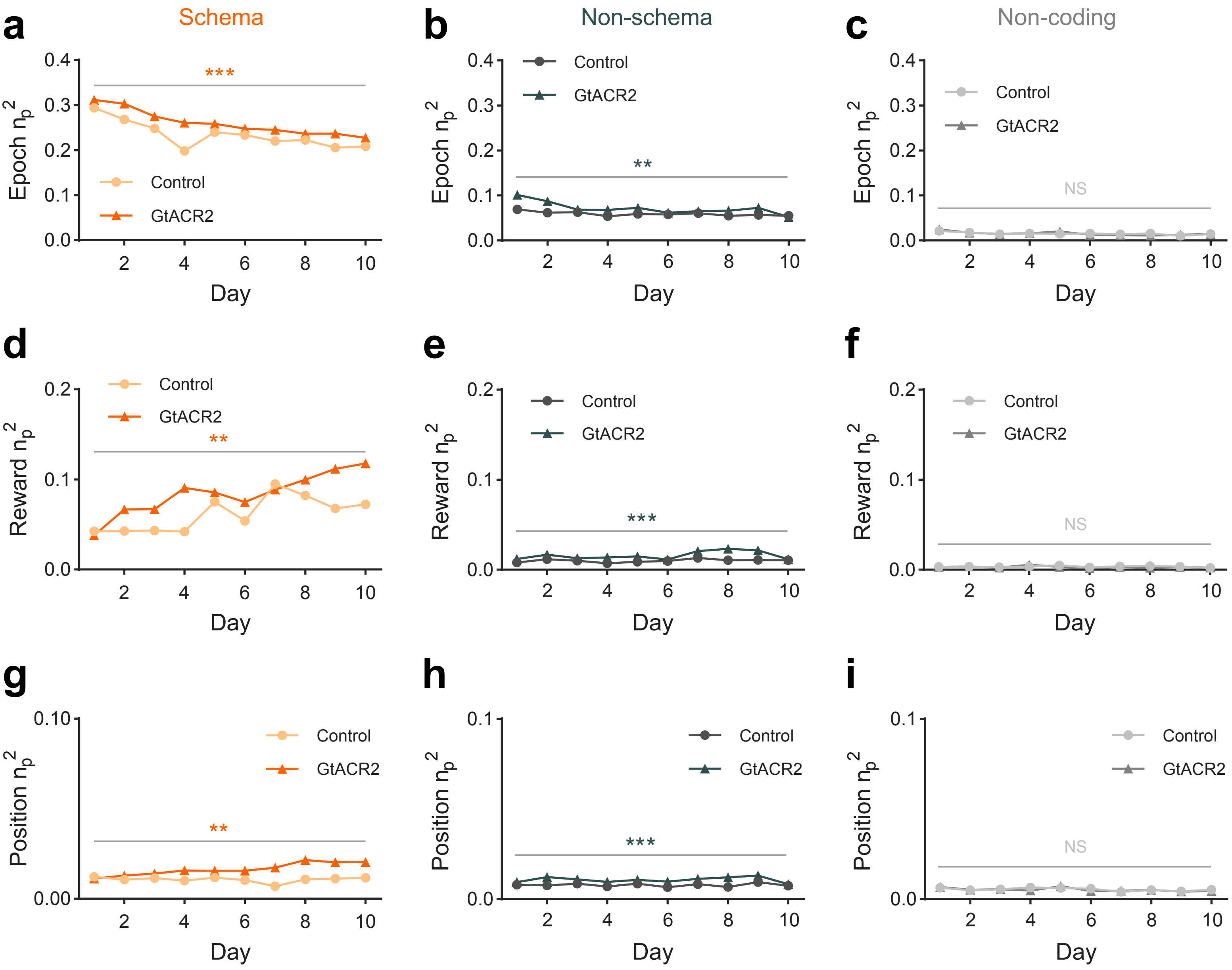

a
Schema
Epoch n_p^2
Control
GtACR2
***
Day
b
Non-schema
Epoch n_p^2
Control
GtACR2
**
Day
c
Non-coding
Epoch n_p^2
Control
GtACR2
NS
Day
d
Reward n_p^2
Control
GtACR2
**
Day
e
Reward n_p^2
Control
GtACR2
***
Day
f
Reward n_p^2
Control
GtACR2
NS
Day
g
Position n_p^2
Control
GtACR2
**
Day
h
Position n_p^2
Control
GtACR2
***
Day
i
Position n_p^2
Control
GtACR2
NS
Day

Supplementary Fig. 9 | Ventral subiculum inactivation increases variance explained by epoch, reward, and position during learning of a new problem. Explained variance, averaged across neurons, is plotted for each neural subpopulation (**a, d, g** - schema, **b, e, h** - non-schema, and **c, f, i** - non-coding) on each day of recording; inactivation resulted in an increase in each type of information in both schema (**a**: $F_{(1,9)} = 33.3$; $P = 2.7 \times 10^{-4}$; **d**: $F_{(1,9)} = 13.3$; $P = 5.4 \times 10^{-3}$; **g**: $F_{(1,9)} = 21.5$; $P = 1.2 \times 10^{-3}$; Two-way ANOVA) and non-schema cells (**b**: $F_{(1,9)} = 13.9$; $P = 4.7 \times 10^{-3}$; **e**: $F_{(1,9)} = 24.4$; $P = 8.0 \times 10^{-4}$; **h**: $F_{(1,9)} = 46.3$; $P = 7.8 \times 10^{-5}$; Two-way ANOVA). No statistically significant differences were observed for any of these variables in non-coding cells ($F < 1.0$; $P > 0.34$; Two-way ANOVA). (***$P < 0.001$; **$P < 0.01$; NS, not significant).

a

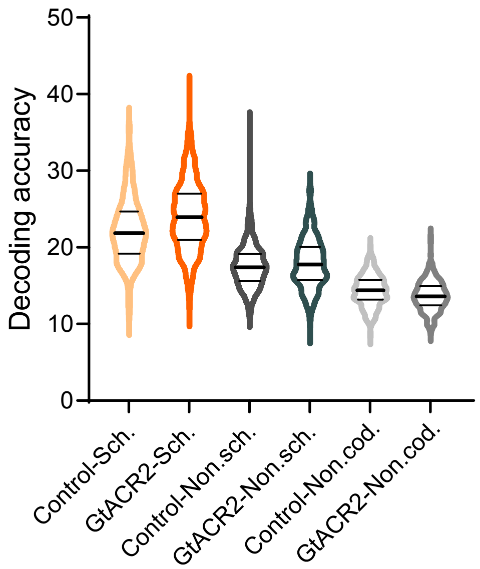

Decoding accuracy
50
40
30
20
10
0
Control-Sch.
GtACR2-Sch.
Control-Non.sch.
GtACR2-Non.sch.
Control-Non.cod.
GtACR2-Non.cod.

Supplementary Fig. 10 | Single-cell decoding performance was assessed for schema, non-schema, and non-coding neurons in the context of learning task. Mean decoding accuracy across all epochs was calculated for each category of cells (**a**). Significant discrepancies were noted among schema, non-schema, and non-coding neurons, evident in both Control ($F_{(1,955)} = 372.2$, $P = 1.0 \times 10^{-15}$, $\eta_p^2 = 0.44$; One-way ANOVA) and GtACR2 groups ($F_{(1,1949)} = 1155.2$, $P = 1.0 \times 10^{-15}$, $\eta_p^2 = 0.54$; One-way ANOVA).

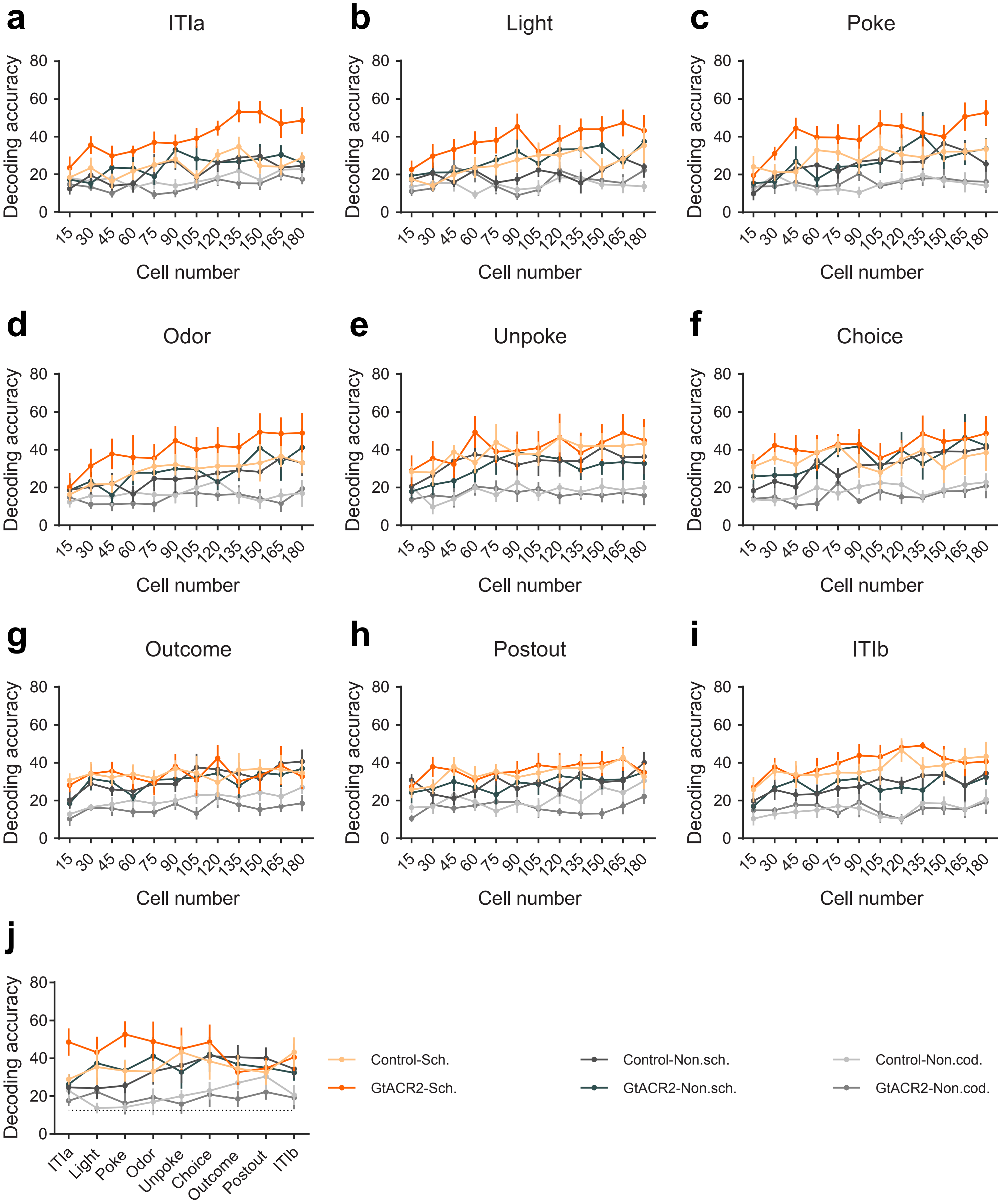

a
ITIa
b
Light
c
Poke
d
Odor
e
Unpoke
f
Choice
g
Outcome
h
Postout
i
ITIb
j
Decoding accuracy
Cell number
15
30
45
60
75
90
105
120
135
150
165
180
ITIa
Light
Poke
Odor
Unpoke
Choice
Outcome
Postout
ITIb
Control-Sch.
GtACR2-Sch.
Control-Non.sch.
GtACR2-Non.sch.
Control-Non.cod.
GtACR2-Non.cod.

Supplementary Fig. 11 | Population level across maze decoding for learning task. Ensembles, including varying numbers of each cell type (**a-i**) and a set of 180 cells across epochs (**j**), were used for this assessment. Significant effects of the three groups (schema, non-schema, and non-coding) were observed in both the Control and GtACR2 groups across each epoch, determined through ANOVA analysis ($P < 1.5 \times 10^{-5}$; Two-way ANOVA). Error bars are SEM.

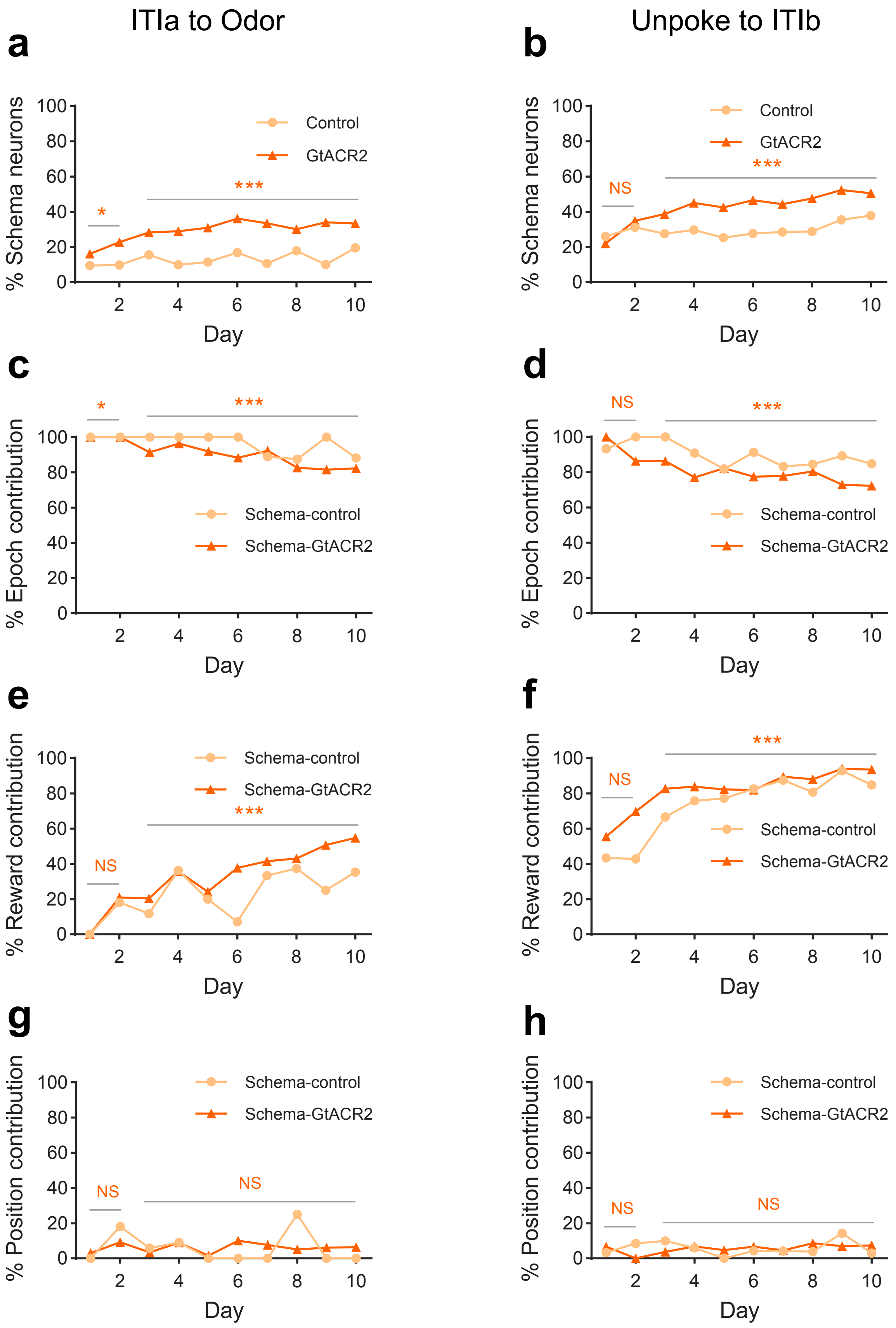
ITIa to Odor
Unpoke to ITIb
a
b
c
d
e
f
g
h
% Schema neurons
% Epoch contribution
% Reward contribution
% Position contribution
Day
Control
GtACR2
Schema-control
Schema-GtACR2
*
***
NS

Supplementary Fig. 12 | Ventral subiculum inactivation facilitates the formation of schema cells in OFC and increases the effect of reward during learning of a new problem, during either early or late parts of the trial. a. Percentage of schema neurons from control and GtACR2 sessions on each day of training at 0.8 threshold, using only data from ITI->odor, prior to external differences related to responding or reward; schema neurons increased more rapidly in the inactivated group (Overall: $\chi^2 = 94.4$, $P = 2.6 \times 10^{-22}$; days 1-2: $\chi^2 = 10.0$; $P = 1.6 \times 10^{-3}$; days 3-10: $\chi^2 = 82.3$, $P = 1.2 \times 10^{-19}$; d.f. = 1; χ^2 test). **b.** same as panel a, but using data from unpoke->ITIb, where response and reward differed; the prevalence of schema neurons was similar initially in both groups and increased more rapidly in the inactivated group (Overall: $\chi^2 = 43.3$, $P = 4.8 \times 10^{-11}$; days 1-2: $\chi^2 = 0.02$; $P = 0.9$; days 3-10: $\chi^2 = 51.7$, $P = 6.3 \times 10^{-13}$; d.f. = 1; χ^2 test). **c-h**, Percentage of schema cells whose correlated activity was affected by shuffling to disrupt information related to epoch (**c-d**), reward (**e-f**), or position (**g-h**) across days of learning. As in the main text, the influence of reward increased as a result of inactivation, both early (**e**: Overall schema: $\chi^2 = 48.2$, $P = 3.9 \times 10^{-12}$; days 1-2: $\chi^2 = 1.6$, $P = 0.2$; days 3-10: $\chi^2 = 44.3$, $P = 2.8 \times 10^{-11}$; d.f. = 1; χ^2 test) and late(**f**: Overall schema: $\chi^2 = 60.2$, $P = 8.6 \times 10^{-15}$; days 1-2: $\chi^2 = 3.5$, $P = 0.062$; days 3-10: $\chi^2 = 54.3$, $P = 1.7 \times 10^{-13}$; d.f. = 1; Chi-squared test) in the trial. In addition, this effect was accompanied by a small but significant decline in the influence of epoch in the inactivated group, again both early (**c**: Overall schema: $\chi^2 = 73.0$, $P = 1.3 \times 10^{-17}$; days 1-2: $\chi^2 = 10.0$, $P = 0.0016$; days 3-10: $\chi^2 = 61.1$, $P = 5.4 \times 10^{-15}$; d.f. = 1; χ^2 test) and late (**d**: Overall schema: $\chi^2 = 14.5$, $P = 1.4 \times 10^{-4}$; days 1-2: $\chi^2 = 0.26$, $P = 0.61$; days 3-10: $\chi^2 = 19.3$, $P = 1.1 \times 10^{-5}$; d.f. = 1; χ^2 test) in the trial, which was not evident in the overall analysis in the main text. (***P < 0.0001; *P < 0.01; NS, not significant).

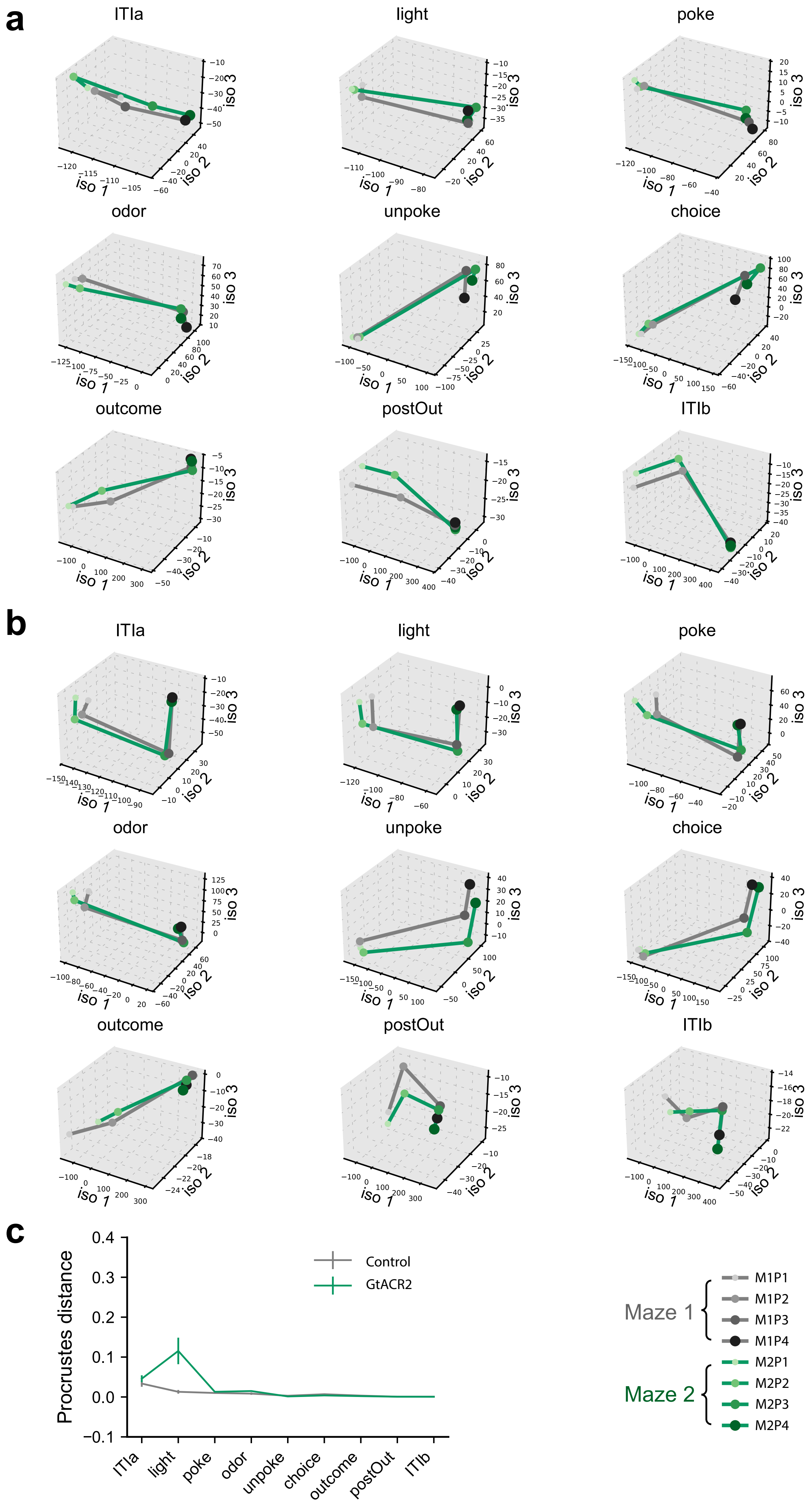
a
ITIa
light
poke
odor
unpoke
choice
outcome
postOut
ITIb
iso 1
iso 2
iso 3
b
ITIa
light
poke
odor
unpoke
choice
outcome
postOut
ITIb
c
Procrustes distance
Control
GtACR2
ITIa
light
poke
odor
unpoke
choice
outcome
postOut
ITIb
Maze 1
M1P1
M1P2
M1P3
M1P4
Maze 2
M2P1
M2P2
M2P3
M2P4

Supplementary Fig. 13 | 3D structure composed of the three leading Isomap components for well-learned task. Positions in Isomap space are illustrated for each maze and epoch in both the Control (**a**) and GtACR2 (**b**) groups during the well-learned task. Gray lines represent Maze 1, while green lines represent Maze 2. For each epoch, the four dots on each line, corresponding to positions P1, P2, P3, and P4, transition from light to dark, with marker sizes increasing progressively from P1 to P4. P1 and P2 represent the common arms, both of which share the same odor, while P3 and P4 correspond to the unique arms, each with distinct odors. **c**, Procrustes analysis was quantified for the Control and GtACR2 groups in (**a**) and (**b**) to assess the geometric similarity between Maze 1 and Maze 2. Across epochs, no significant differences in geometric similarity between the two mazes were observed between the groups (t_{35} = 2.0, P = 0.053; two-tailed Student's t-test; n = 36 for Control and GtACR2). Error bars are SEM.

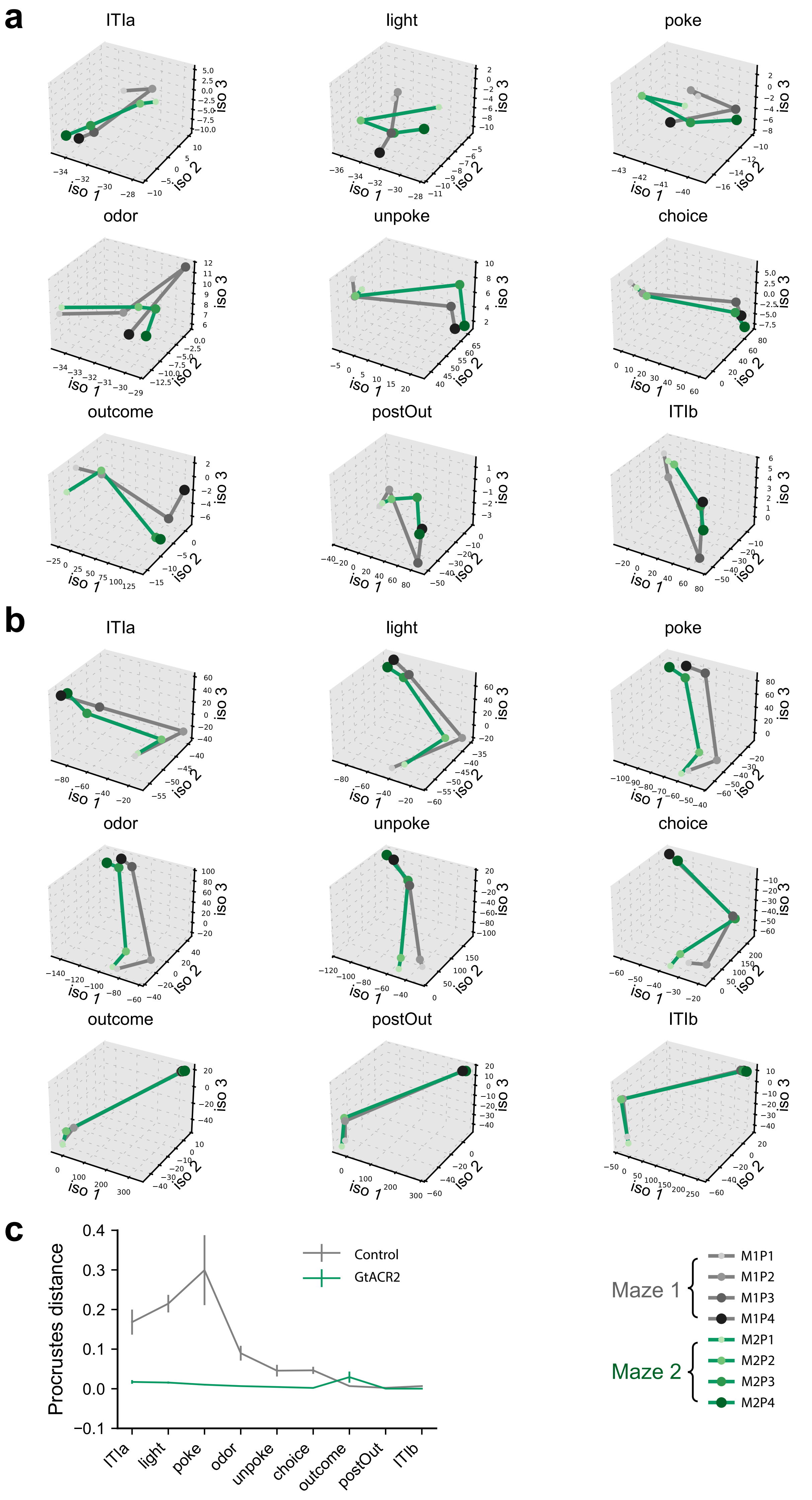

a
ITIa
light
poke
odor
unpoke
choice
outcome
postOut
ITIb
iso 1
iso 2
iso 3
b
ITIa
light
poke
odor
unpoke
choice
outcome
postOut
ITIb
iso 1
iso 2
iso 3
c
Procrustes distance
Control
GtACR2
ITIa
light
poke
odor
unpoke
choice
outcome
postOut
ITIb
Maze 1
M1P1
M1P2
M1P3
M1P4
Maze 2
M2P1
M2P2
M2P3
M2P4

Supplementary Fig. 14 | 3D structure composed of the three leading Isomap components for the learning task. Positions in Isomap space for each maze are illustrated for both the Control (**a**) and GtACR2 (**b**) groups at each learning task epoch. Gray lines denote Maze 1, while green lines represent Maze 2. In each epoch, four dots on each line—corresponding to positions P1, P2, P3, and P4—transition from light to dark, with marker sizes increasing progressively from P1 to P4. P1 and P2 represent the common arms, both sharing the same odor, whereas P3 and P4 correspond to the unique arms, each with distinct odors. **c**, Procrustes analysis was conducted for the Control and GtACR2 groups in (**a**) and (**b**) to assess the geometric similarity between Maze 1 and Maze 2. A significant difference between the two groups was observed (t_{35} = -4.4, P = 9.9 × 10^{-5}; two-tailed Student's t-test; n = 36 for Control and GtACR2). Error bars are SEM.

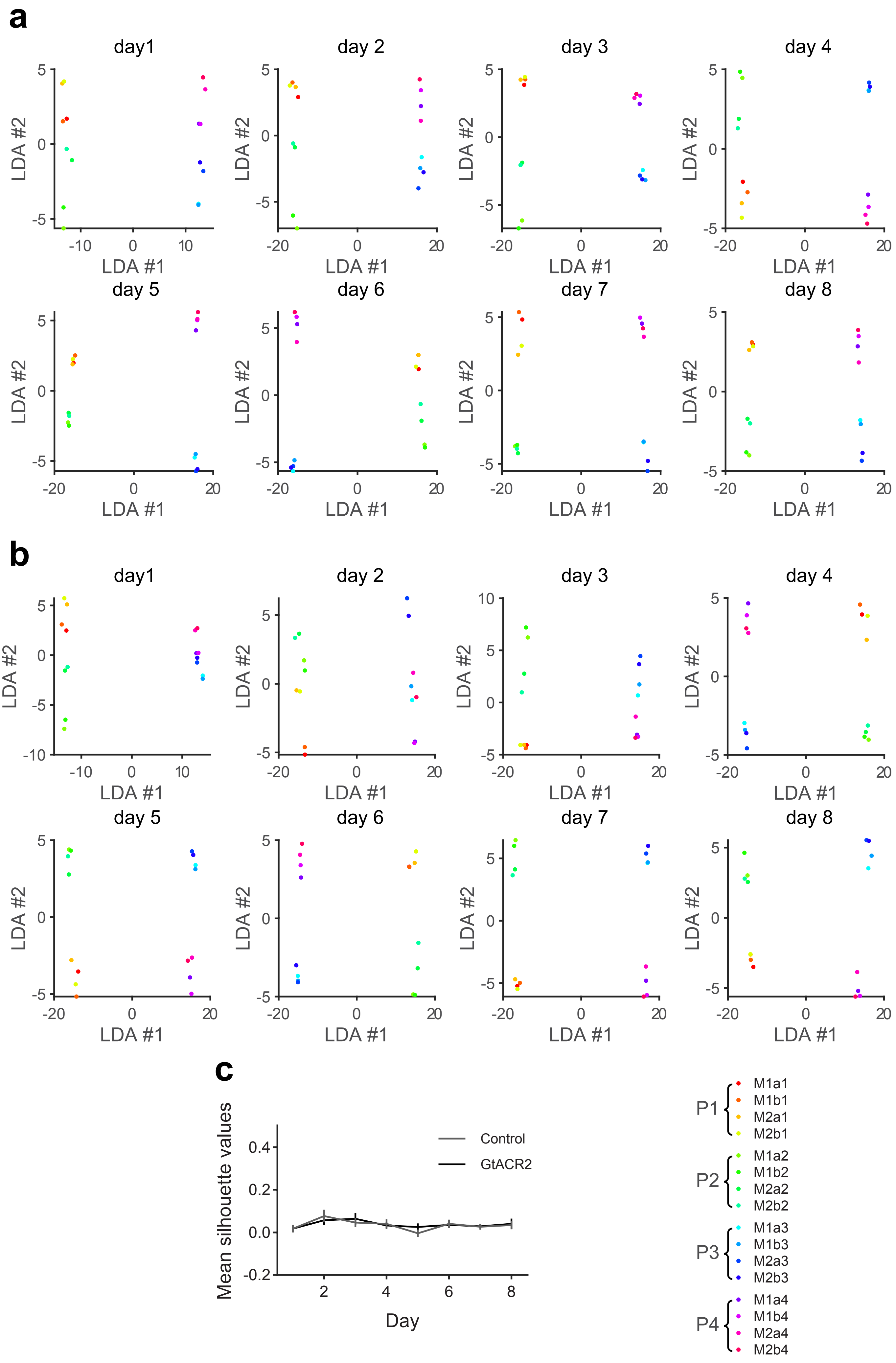
a
day1
day 2
day 3
day 4
day 5
day 6
day 7
day 8
LDA #2
LDA #1
b
day1
day 2
day 3
day 4
day 5
day 6
day 7
day 8
LDA #2
LDA #1
c
Mean silhouette values
Day
Control
GtACR2
P1
M1a1
M1b1
M2a1
M2b1
P2
M1a2
M1b2
M2a2
M2b2
P3
M1a3
M1b3
M2a3
M2b3
P4
M1a4
M1b4
M2a4
M2b4

Supplementary Fig. 15 | LDA clustering plots for the well-learned task across days. Scatter plots of the first two averaged LDAs are presented for the Control (**a**) and GtACR2 (**b**) groups, shown for each day. The task involves 16 trial types, each represented by a distinct colored dot. Ten odors were divided between two mazes (Maze 1 and Maze 2), with each maze further split into two subsequences (a and b), and each subsequence containing four positions (P1–P4). For example, M1a1 indicates Maze 1, sequence a, position 1. Trials from each position clustered together. The positions are classified as P1, P2, P3, and P4, as indicated in the figure legend. **c**, Averaged silhouette values were quantified for each trial type in both the Control and GtACR2 groups during the well-learned task across days. No significant differences were found between the two groups ($t_{14} = 0.28$, $P = 0.79$; two-tailed Student's t-test; n = 8 days for Control and GtACR2). Gray color represents Control group, while the black line represents GtACR2 group. Error bars are SEM.

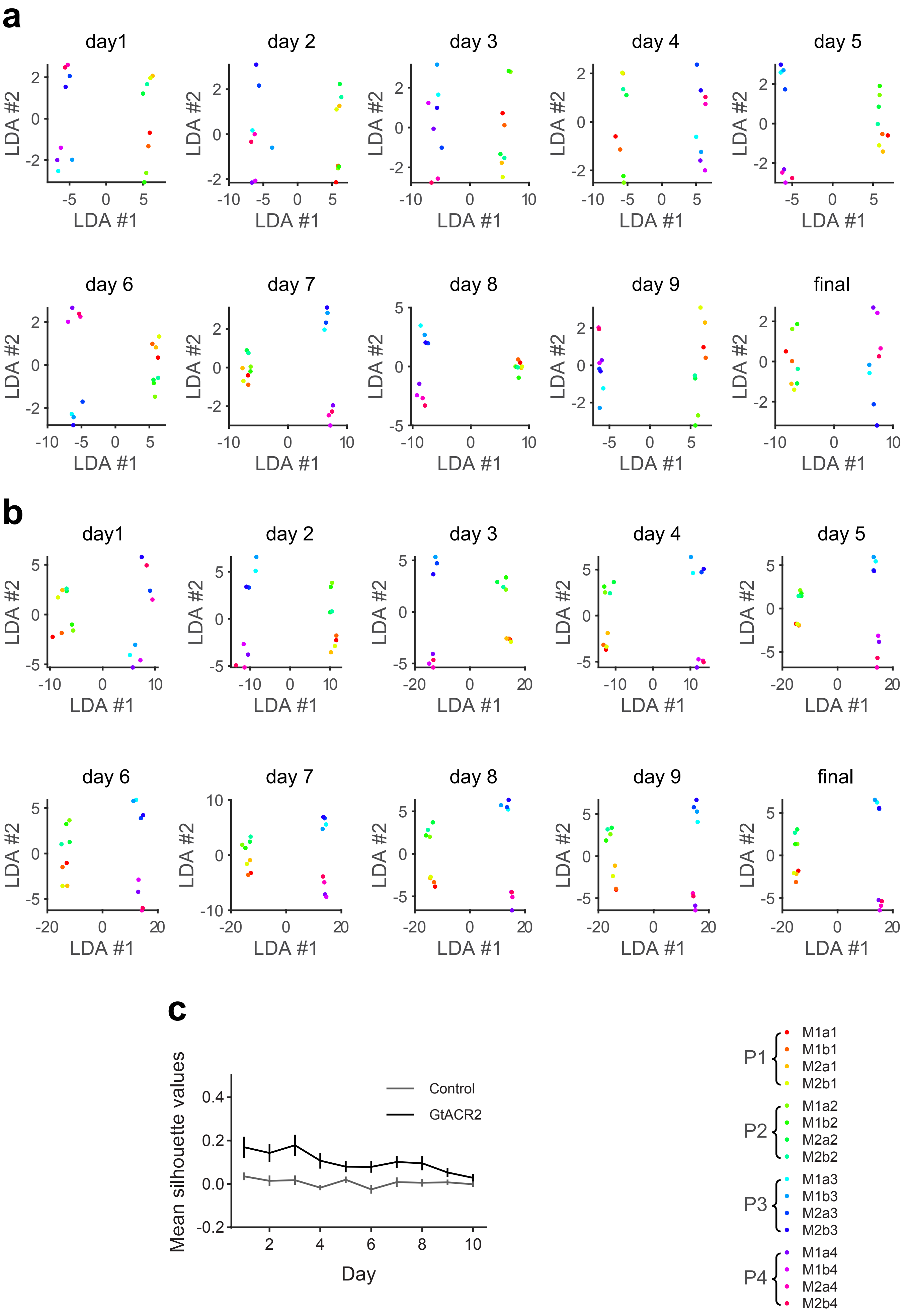
a
day1
day 2
day 3
day 4
day 5
day 6
day 7
day 8
day 9
final
LDA #1
LDA #2
b
day1
day 2
day 3
day 4
day 5
day 6
day 7
day 8
day 9
final
LDA #1
LDA #2
c
Mean silhouette values
Day
Control
GtACR2
P1
M1a1
M1b1
M2a1
M2b1
P2
M1a2
M1b2
M2a2
M2b2
P3
M1a3
M1b3
M2a3
M2b3
P4
M1a4
M1b4
M2a4
M2b4

Supplementary Fig. 16 | LDA clustering plots for the learning task across days. Scatter plots of the first two averaged LDAs are shown for the Control (**a**) and GtACR2 (**b**) groups. The task consists of 16 trial types, each represented by a distinct colored dot. Ten odors were distributed between two mazes (Maze 1 and Maze 2), with each maze further divided into two subsequences (a and b), and each subsequence comprising four positions (P1–P4). For example, M1a1 refers to Maze 1, sequence a, position 1. Trials from each position clustered together. The positions are classified as P1, P2, P3, and P4, as indicated in the figure legend. **c**. Averaged silhouette values were calculated for each trial type in both the Control and GtACR2 groups during the learning task across days. The GtACR2 group showed higher silhouette values compared to the Control group (t_{18} = -5.9, P = 1.3 × 10^{-5}; two-tailed Student's t-test; n = 8 days for Control and GtACR2). Gray lines represent the Control group, while black lines represent the GtACR2 group. Error bars indicate SEM.

a

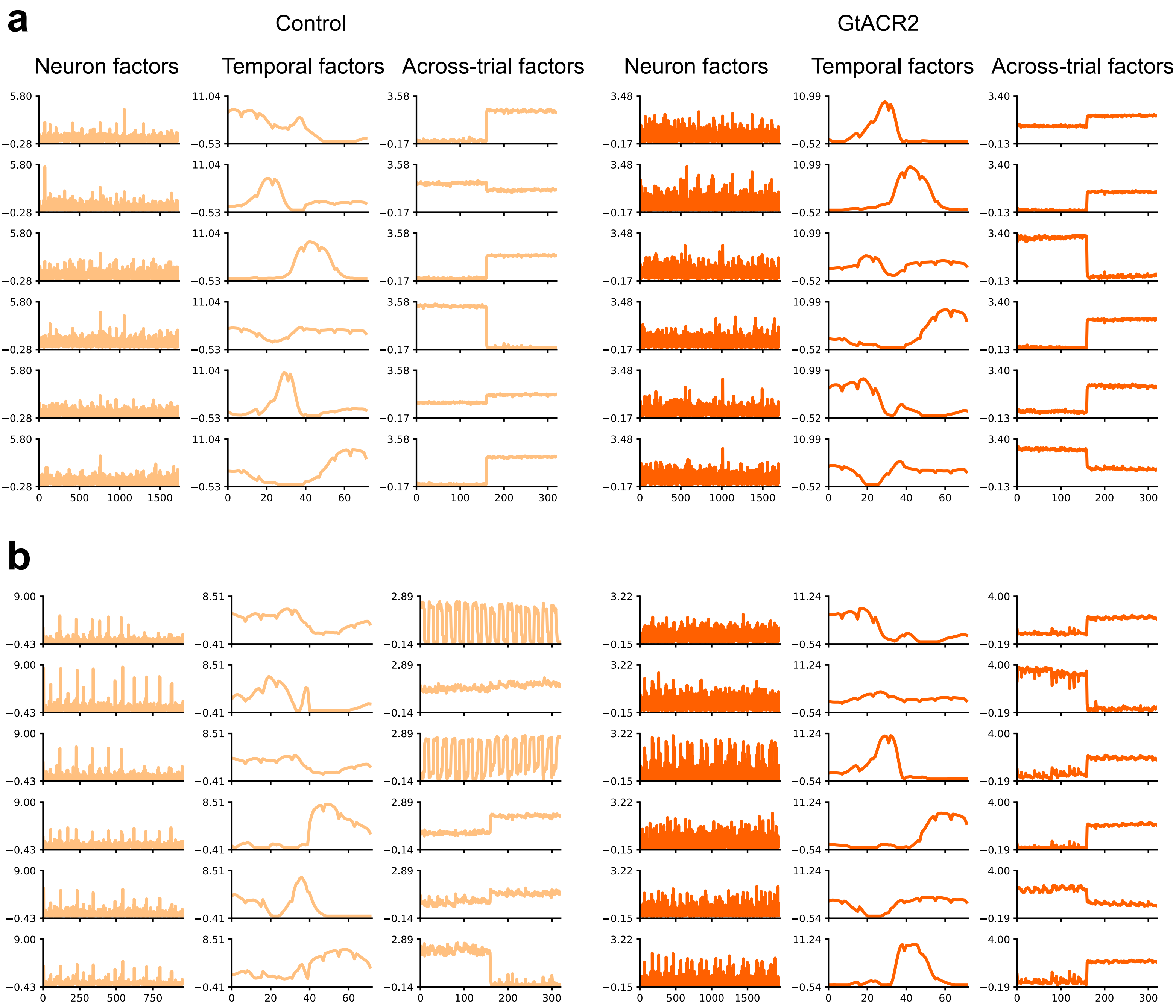
a
Control
GtACR2
Neuron factors
Temporal factors
Across-trial factors
Neuron factors
Temporal factors
Across-trial factors
b

Supplementary Fig. 17 | Six components of non-negative TCA of OFC neuron activity are shown. Each component consists of a neuron factor (left column), a temporal factor across 9 events and 8 time points per event (middle column), and a trial factor (right column). Temporal factors are grouped as follows: factors 1–8 correspond to ITIa, factors 9–16 to Light, factors 17–24 to Poke, factors 25–32 to Odor, factors 33–40 to Unpoke, factors 41–48 to Choice, factors 49–56 to Outcome, factors 57–64 to Postout, and factors 65–72 to ITIb. There are 16 trial types, with 20 trials for each type. Trials 1–160 are negative trials, while trials 161–320 are positive trials. These low-dimensional components are derived from a 6-component model for both the well-learned task (**a**) and the learning task (**b**). **a**, All components reveal neurons encoding trial outcomes in both groups for the well-learned task. In the Control group, components 1, 3, 5, and 6 encode positive trials, while components 2 and 4 encode negative trials. Similarly, in the GtACR2 group, components 1, 2, 4, and 5 encode positive trials, and components 3 and 6 encode negative trials. **b,** In the learning task for the GtACR2 group, the components also encode trial outcomes similar to the well-learned task, with components 1, 3, 4, and 6 encoding positive trials, and components 2 and 5 encoding negative trials. However, in Control group, in addition to components 4 and 5 encoding positive trials, component 6 encodes negative trials. We also identified other components that specifically encode each trial type (components 1 and 3) as well as early components that are active across nearly all trials (component 2).

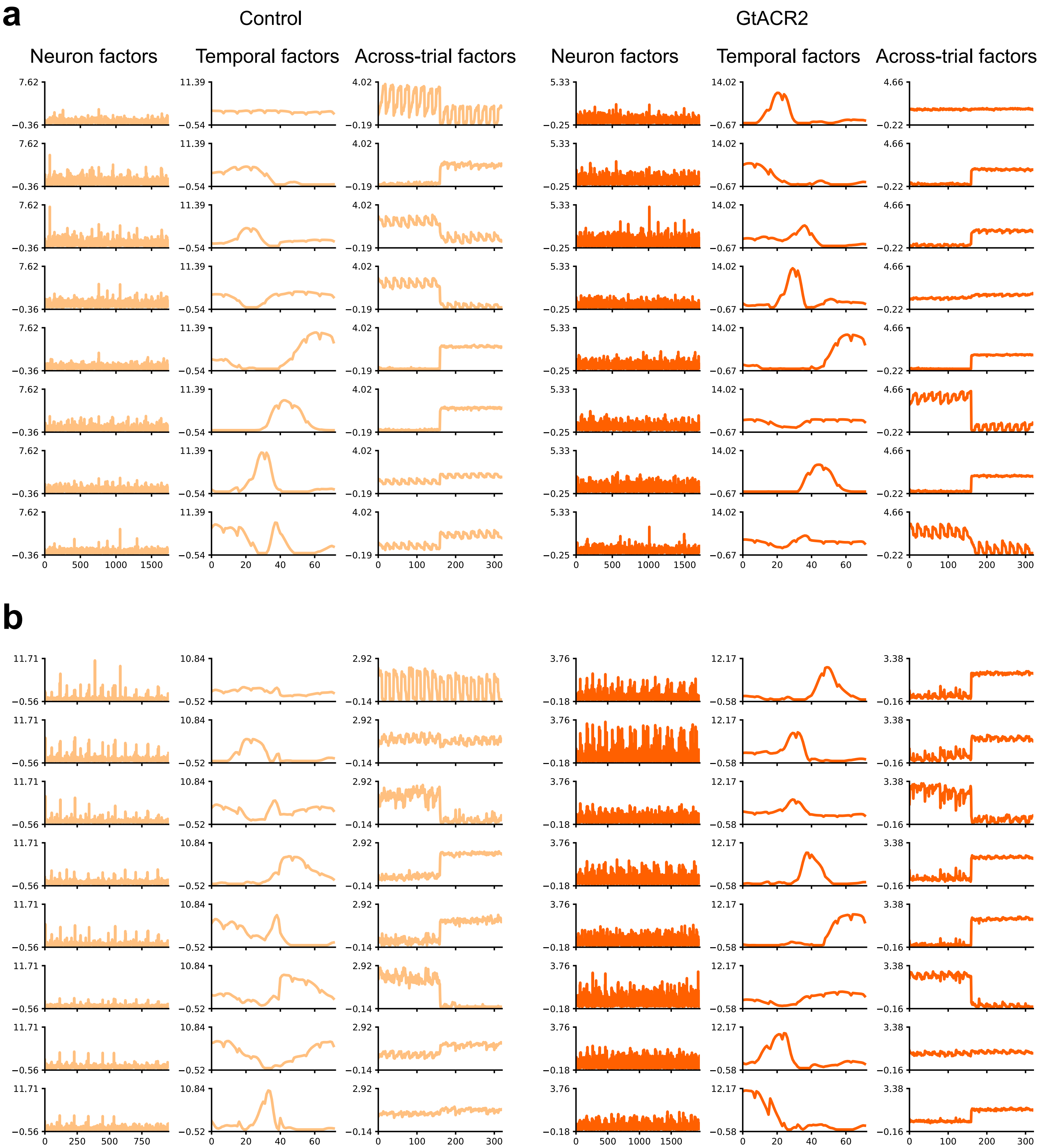
a
Control
GtACR2
Neuron factors
Temporal factors
Across-trial factors
Neuron factors
Temporal factors
Across-trial factors
b

Supplementary Fig. 18 | Eight components of nonnegative TCA of OFC neuron activity are depicted. Each component consists of a neuron factor (left column), a temporal factor across 9 events and 8 time points per event (middle column), and a trial factor (right column). Temporal factors are grouped as follows: factors 1–8 correspond to ITIa, 9–16 to Light, 17–24 to Poke, 25–32 to Odor, 33–40 to Unpoke, 41–48 to Choice, 49–56 to Outcome, 57–64 to Postout, and 65–72 to ITIb. There are 16 trial types, with 20 trials for each type. Trials 1–160 are negative, while trials 161–320 are positive. These low-dimensional components are derived from an 8-component model for both the well-learned task (**a**) and the learning task (**b**). **a**, Most components reveal neurons encoding trial outcomes in both groups for the well-learned task. In the Control group, components 2, 5, 6, 7, and 8 encode positive trials, while components 1, 3, and 4 encode negative trials. Similarly, in the GtACR2 group, components 2, 3, 4, 5, and 7 encode positive trials, components 6 and 8 encode negative trials, while component 1 shows early component patterns. **b**, In the learning task for the GtACR2 group, components also encode trial outcomes similar to the well-learned task. Components 1, 2, 4, 5, and 8 encode positive trials, components 3 and 6 encode negative trials, and component 7 represents early components. In the Control group, components 4, 5, and 7 encode positive trials, while components 3 and 6 encode negative trials. Additionally, other components were identified that specifically encode each trial type (component 1), as well as components that encode both trial types and early events (components 2 and 8).
