# Supplementary material for: Hippocampal output suppresses orbitofrontal cortex schema cell formation
Source: Nat Neurosci. 2025 Apr 14;28(5):1048–60. doi: 10.1038/s41593-025-01928-z (PMC12081290; doi:10.1038/s41593-025-01928-z)
Supplement: Supplementary file 2 — Reporting Summary [file 41593_2025_1928_MOESM2_ESM.pdf]

nature portfolio

Corresponding author(s): Geoffrey Schoenbaum, Wenhui Zong

Last updated by author(s): Jan 22, 2025

Reporting Summary

Nature Portfolio wishes to improve the reproducibility of the work that we publish. This form provides structure for consistency and transparency in reporting. For further information on Nature Portfolio policies, see our Editorial Policies and the Editorial Policy Checklist.

Statistics

For all statistical analyses, confirm that the following items are present in the figure legend, table legend, main text, or Methods section.

n/a	Confirmed	
☐	☒	The exact sample size (*n*) for each experimental group/condition, given as a discrete number and unit of measurement
☐	☒	A statement on whether measurements were taken from distinct samples or whether the same sample was measured repeatedly
☐	☒	The statistical test(s) used AND whether they are one- or two-sided<br>*Only common tests should be described solely by name; describe more complex techniques in the Methods section.*
☐	☒	A description of all covariates tested
☐	☒	A description of any assumptions or corrections, such as tests of normality and adjustment for multiple comparisons
☐	☒	A full description of the statistical parameters including central tendency (e.g. means) or other basic estimates (e.g. regression coefficient) AND variation (e.g. standard deviation) or associated estimates of uncertainty (e.g. confidence intervals)
☐	☒	For null hypothesis testing, the test statistic (e.g. *F*, *t*, *r*) with confidence intervals, effect sizes, degrees of freedom and *P* value noted<br>*Give P values as exact values whenever suitable.*
☒	☐	For Bayesian analysis, information on the choice of priors and Markov chain Monte Carlo settings
☒	☐	For hierarchical and complex designs, identification of the appropriate level for tests and full reporting of outcomes
☐	☒	Estimates of effect sizes (e.g. Cohen's *d*, Pearson's *r*), indicating how they were calculated

Our web collection on statistics for biologists contains articles on many of the points above.

Software and code

Policy information about availability of computer code

Data collection	Spikes and behavioral data were collected using Plexon OmniPlex system, spike sorting using Plexon Offline Sorter x64 v4.0.
Data analysis	Data analysis was performed using custom MATLAB code(MATLAB R2021b), liblinear-master, and Spyder 6. TensorTools package developed by Williams et al., 2018, for analysis. All scripts used in this study are available at https://osf.io/78gy4/.

For manuscripts utilizing custom algorithms or software that are central to the research but not yet described in published literature, software must be made available to editors and reviewers. We strongly encourage code deposition in a community repository (e.g. GitHub). See the Nature Portfolio guidelines for submitting code & software for further information.

Data

Policy information about availability of data

All manuscripts must include a data availability statement. This statement should provide the following information, where applicable:

- Accession codes, unique identifiers, or web links for publicly available datasets
- A description of any restrictions on data availability
- For clinical datasets or third party data, please ensure that the statement adheres to our policy

The dataset used in this study are available at https://osf.io/78gy4/.

Research involving human participants, their data, or biological material

Policy information about studies with human participants or human data. See also policy information about sex, gender (identity/presentation), and sexual orientation and race, ethnicity and racism.

Reporting on sex and gender	The study did not involve human participants.
Reporting on race, ethnicity, or other socially relevant groupings	n/a
Population characteristics	n/a
Recruitment	n/a
Ethics oversight	n/a

Note that full information on the approval of the study protocol must also be provided in the manuscript.

Field-specific reporting

Please select the one below that is the best fit for your research. If you are not sure, read the appropriate sections before making your selection.

☒ Life sciences ☐ Behavioural & social sciences ☐ Ecological, evolutionary & environmental sciences

For a reference copy of the document with all sections, see nature.com/documents/nr-reporting-summary-flat.pdf

Life sciences study design

All studies must disclose on these points even when the disclosure is negative.

Sample size	The number of rats and neurons was not predetermined using statistical methods but aligns with those reported in previous studies from our lab and others in the field. For each task, units were collected from at least three rats per group. Specifically, over 1,800 units were recorded for the well-learned task, while more than 800 units were collected and analyzed for the learning task.
Data exclusions	No animals were excluded from analysis.
Replication	Subsets of animals and neurons were randomly drawn from the whole dataset for cross-validation to avoid over-fitting. To account for unequal sample sizes when comparing preferred versus non-preferred signals, we drew equal-sized bootstrapped data samples and calculated a bootstrap statistic for each trial type using MATLAB's 'bootstrp' function. Statistical significance was assumed for P<0.05. All replicates were successful.
Randomization	Rats were randomly divided into control and inactivation groups, both groups received same training.
Blinding	The investigators were blinder to group allocation during data collection and/or analysis.

Reporting for specific materials, systems and methods

We require information from authors about some types of materials, experimental systems and methods used in many studies. Here, indicate whether each material, system or method listed is relevant to your study. If you are not sure if a list item applies to your research, read the appropriate section before selecting a response.

Materials & experimental systems

n/a	Involved in the study	
☐	☒	Antibodies
☒	☐	Eukaryotic cell lines
☒	☐	Palaeontology and archaeology
☐	☒	Animals and other organisms
☒	☐	Clinical data
☒	☐	Dual use research of concern
☒	☐	Plants

Methods

n/a	Involved in the study	
☒	☐	ChIP-seq
☒	☐	Flow cytometry
☒	☐	MRI-based neuroimaging

Antibodies

Antibodies used	1. Product: Anti-RFP antibody; supplier name: evrogen; CAT.#: AB233;<br>2. Product: Alexa Fluor® 647 AffiniPure F(ab')$_2$ Fragment Donkey Anti-Rabbit IgG (H+L); supplier: Jackson ImmunoResearch; catalog number; 711-605-152.
Validation	The antibody has been selected to recognize both denatured and native TurboRFP, TurboFP602, TurboFP635, Katushka2S, TurboFP650, NirFP, TagBFP, TagRFP, FusionRed, TagFP635, mKate2, PA-TagRFP, mRuby and mCherry. - Superior performance in fusions<br>- Low cytotoxicity<br>- Fast maturation, high pH-stability and photostability<br>- Proven suitability to generate stably transfected cell lines<br>- Recommended for protein labeling and long-term experiments. Detailed information can be found here: https://evrogen.com/products/FusionRed/FusionRed_Detailed_description.shtml.

Animals and other research organisms

Policy information about studies involving animals; ARRIVE guidelines recommended for reporting animal research, and Sex and Gender in Research

Laboratory animals	Subjects were four female and four male Long-Evans rats (Charles River Laboratories, 160-300 g) aged ~3 months at the start of the experiment.
Wild animals	The study did not use any wild animals.
Reporting on sex	Equal number of male and female rats were used. I do not expect to find significant sex differences,<br>and thus plan to use an equal number of males and females in each group.
Field-collected samples	The study did not involve field-collected samples.
Ethics oversight	All behavioral testing was carried out at the NIDA-IRP. Animal care and experimental procedures complied with the U.S. National Institutes of Health (NIH) guidelines and were approved by the Animal Care and Use Committee (ACUC) at the NIDA-IRP.

Note that full information on the approval of the study protocol must also be provided in the manuscript.

Plants

Seed stocks	The study did not involve human participants.
Novel plant genotypes	n/a
Authentication	n/a
